# Anti-hypertensive medication adherence, socioeconomic status, and cognitive aging in the Chinese community-dwelling middle-aged and older adults ≥ 45 years: a population-based longitudinal study

**DOI:** 10.1186/s12916-025-03949-8

**Published:** 2025-02-25

**Authors:** Chenglong Li, Daijun He, Yufan Liu, Chao Yang, Luxia Zhang

**Affiliations:** 1 National Institute of Health Data Science at Peking University, Beijing, 100191 China; 2 Institute of Medical Technology, Health Science Center of Peking University, Beijing, 100191 China; 3 Renal Division, Department of Medicine, Peking University First Hospital, 8 Xishiku Street, Xicheng District, Beijing, 100034 China; 4 Institute of Nephrology, Key Laboratory of Renal Disease, Ministry of Health of China and Key Laboratory of Chronic Kidney Disease Prevention and Treatment (Peking University), Ministry of Education, Beijing, China; 5 Research Units of Diagnosis and Treatment of Immune-mediated Kidney Diseases, Chinese Academy of Medical Sciences, Beijing, China; 6 Capital Medical University, Beijing, China; 7 Advanced Institute of Information Technology, Peking University, Hangzhou, 311215 China; 8 State Key Laboratory of Vascular Homeostasis and Remodeling, Peking University, Beijing, 100191 China

**Keywords:** Anti-hypertensive medication, Hypertension, Cognitive decline, Dementia

## Abstract

**Background:**

It remains unclear whether anti-hypertensive medication use is associated with cognitive aging in general Chinese middle-aged and older adults, as well as the interplay with socioeconomic status (SES). We aim to examine associations of anti-hypertensive medication adherence, SES, and cognitive aging in Chinese middle-aged and older adults.

**Methods:**

Our study was based on the China Health and Retirement Longitudinal Study, an ongoing longitudinal national survey recruiting community-dwelling adults aged ≥ 45 years. Baseline anti-hypertensive medication use was assessed at wave 1. Longitudinal adherence to anti-hypertensive medication was assessed during waves 1 and 2. SES was assessed using income, education, employment, and medical insurance. The annual rate of cognitive change was assessed using cognitive Z scores. Linear mixed models were used to examine longitudinal associations.

**Results:**

A total of 9229 participants were included (mean [SD] age: 57.1 [8.9] years; men: 50.8%). After controlling for blood pressure and other characteristics, participants taking anti-hypertensive medication at baseline, compared to participants not using medication, had a significantly decelerated decline in global cognition (β = 0.014; 95% confidence interval [CI], 0.003 to 0.025 SD/year; *P* = 0.01) and memory (β = 0.021; 95% CI, 0.008 to 0.034 SD/year; *P* = 0.001), respectively. Similarly, participants with high anti-hypertensive medication adherence during follow-up had slower declines in global cognition (β = 0.014; 95% CI, 0.002 to 0.027 SD/year; *P* = 0.02) and memory (β = 0.023; 95% CI, 0.008 to 0.038 SD/year; *P* = 0.003), compared to the low adherence group. There were no significant differences in cognitive decline between hypertension participants using or persistently adhering to medication and normotension participants. The SES significantly interacted with anti-hypertensive medication in associations with cognitive aging, with more evident associations observed in low SES subgroup (all *P* for interaction < 0.05). Several sensitivity analyses were conducted, observing consistent findings.

**Conclusions:**

Adhering to anti-hypertensive medication was associated with decelerated cognitive aging in Chinese community-dwelling middle-aged and older adults, especially in participants with low SES. These findings indicate that promoting anti-hypertensive medication use could be important to achieve healthy and inclusive cognitive aging in general Chinese middle-aged and older adults living with hypertension.

**Supplementary Information:**

The online version contains supplementary material available at 10.1186/s12916-025-03949-8.

## Background

Dementia has become a non-neglectable public health challenge, accounting for substantial proportions of mortality and disability around the world [1]. According to the World Alzheimer Report 2023, the number of people living with dementia in the world is expected to increase from 55 million in 2019 to 139 million in 2050, with annual costs reaching 2.8 trillion dollars by 2030 [2]. WHO has listed risk reduction as one crucial aspect of the global dementia action plan [3], necessitating further investigations targeting modifiable risk factors to promote primary prevention.

Hypertension is one modifiable risk factor for dementia. According to the Lancet Commission Report 2020 on Dementia Prevention, Intervention, and Care, midlife (age 45 to 65 years) hypertension accounts for 1.9% of dementia cases globally [1]. In low-income and middle-income countries, the proportion of dementia attributable to midlife hypertension ranges from 4 to 9% [4]. Our previous work reveals associations between elevated blood pressure (BP) since midlife with subsequent cognitive decline and dementia onset [5].

So far, both observational and interventional studies have found that controlling hypertension could be beneficial in preventing dementia onset or cognitive decline. The well-renowned Systolic Blood Pressure (SBP) Intervention Trial (SPRINT) confirmed intensive SBP control of less than 120 mmHg, compared to 140 mm Hg, achieved a 15% reduced risk in the combined outcome of mild cognitive impairment or probable dementia [6]. At the same time, no statistically significant protective effect was observed for probable dementia alone [6]. A meta-analysis of five randomized trials in patients aged > 65 years found antihypertensive treatment use reduced dementia risk by 13% after a median follow-up of 4.3 years, with a mean SBP/diastolic blood pressure (DBP) lowering of 10/4 mm Hg [7]. Findings from observational studies also suggested relationships between anti-hypertensive medication and reduced dementia risk [8, 9].

Despite inspiring advances, few investigations have examined associations between anti-hypertensive medication adherence, especially longitudinal adherence, with long-term cognitive aging rate in general middle-aged and older adults. There are other research gaps requiring further investigations. First, current available trials are based on highly selected clinical population, with limited implications for the general population. Second, accelerated cognitive decline is regarded as an important indicator of the preclinical stage of dementia [10]. Nevertheless, most previous studies have exclusively evaluated dementia onset, rarely accounting for the longitudinal rate of cognitive aging. Third, evidence is lacking in the general Chinese middle-aged and older adults [11], which had both a high prevalence of undiagnosed hypertension and a high proportion of dementia burden attributable to midlife hypertension [4, 12]. Finally, socioeconomic status (SES) is a crucial and significant determinant of healthy aging [13]. It has been found the lower SES contributed to acceleration of aging in multiple facets, including physical, sensory, physiological, cognitive, emotional, and social domains, independently from other factors [14]. Previous studies also showed SES was associated with inequalities in dementia onset [15]. Moreover, the SES disparities impact the adherence to anti-hypertensive medication, indicating the necessity of comprehensively exploring the interplay between the two factors [16]. So far, few studies have comprehensively examined the role of SES in associations between anti-hypertensive medication and cognitive aging.

Therefore, we intend to investigate longitudinal associations between anti-hypertensive medication use and subsequent cognitive decline, based on a large, nationally representative cohort of Chinese middle-aged and older adults. We considered both baseline medication status and adherence to medication during follow-up. We also evaluated joint associations of anti-hypertensive medication use and SES on cognitive aging.

## Methods

### Study population

The current study is based on the China Health and Retirement Longitudinal Study (CHARLS), an ongoing national longitudinal survey recruiting community-dwelling middle-aged and older adults ≥ 45 years, covering more than 10,000 households and 17,000 individuals in 150 counties/districts and 450 villages/resident committees across 28 provinces in China. A multistage probability sampling was applied for selecting participants for enrollment at each survey. Several sampling techniques were applied to ensure the representativeness, including probability-proportional-to-size sampling, stratified sampling by region/districts and per capita statistics on gross domestic product, etc. Further details about CHARLS’s objectives, design (including the sampling framework), and methods can be found elsewhere [17]. The CHARLS conducted surveys at regular time intervals, which is called waves. Data is routinely collected, including sociodemographic characteristics, lifestyle factors, physical examination measurements, clinical conditions and treatment, as well as cognitive function. The baseline survey, e.g. wave 1, began in 2011, with subsequent waves conducted every two or three years, including wave 2 (2013), wave 3 (2015), and wave 4 (2018). We used the 7-year survey data for research, with the wave 1 survey regarded as the baseline. The CHARLS was approved by the Peking University Institutional Review Board (IRB00001052-11015). Before being included, all participants provided informed consent.

**Fig. 1:**
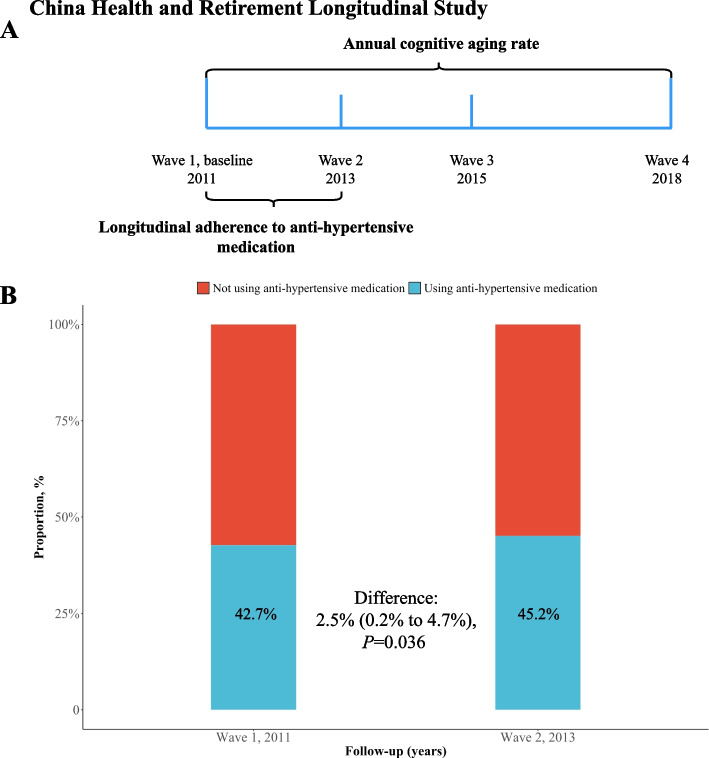
Study timeline and anti-hypertensive medication time course. **A** Study timeline. **B** Proportion of anti-hypertensive medication use during wave 1 and wave 2, restricted to participants with hypertension at baseline. The difference in proportion of medication use between wave 1 and wave 2 was examined using the chi-squared test, with asymptotic standard errors and 95% Wald confidence limits calculated

Among the 17,708 participants attending wave 1 of CHARLS, we further excluded 175 participants without basic demographic information (age and sex), 326 participants reported memory-related disease at wave 1, 3778 participants without BP measurements at wave 1, 3015 participants without cognitive function measurements at wave 1, and 1185 participants without cognitive function re-assessments during waves 2 to 4. Finally, 9229 participants were included for analysis, with a detailed selection procedure shown in Additional file 1: Fig. S1. This study followed the Strengthening the Reporting of Observational Studies in Epidemiology (STROBE) reporting guideline. The study timeline and design are presented in Fig. 1A.

### Hypertension and anti-hypertensive medication

At baseline, both SBP and DBP were measured by trained research staff three times at 45-s intervals, using the Omron digital devices (Omron™ HEM-7200 Monitor) [17]. Aligning with a previous study [12], we used the average of the second and third BP readings to determine the BP level. For hypertension diagnosis, participants were asked whether a doctor had told them he/she had hypertension, with further confirmation conducted by research staff. According to the 2017 American College of Cardiology/American Heart Association hypertension guidelines [18], we defined hypertension as a physician-confirmed diagnosis or mean SBP/DBP ≥ 140/90 mm Hg or taking antihypertensive medication.

Participants were asked whether they used any Western modern medicine to control hypertension. And those answering “yes” were categorized as using anti-hypertensive medication at the current wave. Based on hypertension status and anti-hypertensive medication use at baseline, we categorized baseline medication use as 1) normotension, without using medication; 2) hypertension not using anti-hypertensive medication; and 3) hypertension using anti-hypertensive medication. Based on baseline and wave 2, we categorized longitudinal adherence to anti-hypertensive medication using the number of waves reporting anti-hypertensive medication use, including 1) normotension, without using medication; 2) hypertension with low adherence (not using anti-hypertensive medication in both waves 1 and 2, e.g. using medication for 0 wave); 3) hypertension with moderate adherence (using anti-hypertensive medication in single wave 1 or 2, e.g. using medication for 1 wave); and 4) hypertension with high adherence (using anti-hypertensive medication in both waves 1 and 2, e.g. using medication for 2 waves).

### Socioeconomic status

We considered four indicators of SES, including family annual income, education, employment status, and medical insurance coverage, identical to our previous work [19]. Family annual income was assessed as the total income at the household level for the last calendar year of each participant. Then, based on the median level ($2877.7, equivalent to 18130.0 in CNY), participants were further categorized into annual income above the median and below the median, respectively. Education was categorized into three levels, including less than high school, high school or equivalent (general educational development), and college or above college. Employment status was grouped as employed (including participants with paid employment or self-employed, as well as retired) and unemployed. Medical insurance coverage was categorized into insured (covered by private health insurance plans or public health insurance plans) and uninsured.

In line with a previous study [20], we also used the latent class analysis (LCA) to generate an overall SES variable, based on above SES indicators. The LCA uses multiple observed categorical variables to derive a latent variable with mutually exclusive latent classes. After assessing model fitting statistics and referencing from the previous study [20], a latent SES variable with two classes was eventually identified based on item-response probabilities, representing subpopulations of the lower and upper SES, respectively. The item response probabilities of identified latent classes were presented in Additional file 1: Table S1. We also compared the distribution of SES indicators between SES classes, supporting the validity of identified SES classes (Additional file 1: Table S2).

### Cognitive function

The outcome of the present study was the annual rate of cognitive aging, estimated using repeated cognitive function measurements from surveys of CHARLS. Standardized cognitive batteries of different cognitive domains were administered to assess cognition function at each wave of the CHARLS cohort, including memory function, executive function, and temporal orientation [21]. Further details regarding administered cognitive batteries and scoring criteria were presented in Additional file 1: Supplementary Methods. All cognitive batteries have been applied by previous studies, with a higher score indicating better cognitive performance [5, 12, 22, 23].

To reflect the annual rate of cognitive aging and enable comparison across test batteries, standardized cognitive Z scores were calculated. First, we calculated Z scores for each of the three cognitive domains (memory, executive function, and orientation), using the mean and standard deviation (SD) of raw cognitive scores at baseline. Then, we calculated the global cognition Z score by averaging the Z scores for the three cognitive domains and re-standardizing the global Z score using the mean and SD of the baseline global cognitive Z scores. Similar approaches for handling raw cognitive scores have been well-embraced in previous studies [5, 12, 22, 23]. For the calculated Z score, a value of −1 at any given wave indicated that the cognitive score was 1 SD below the mean cognitive score at baseline. Both the global and domain-specific cognitive Z scores were analyzed, to reflect the annual rate of cognitive aging in global and sub-domains of cognition, expressed as SD/year.

### Covariates

Based on previous studies [5, 12], we selected covariates for adjustment, all of which were assessed at baseline. Sociodemographic characteristics included age (years), sex, educational level, and cohabitation status (living alone or not). Lifestyle factors included physical activity (engaging in vigorous or moderate activities no less than once weekly), alcohol consumption (at least once per week), and current smoking (yes or no). Clinical characteristics included physical disability and reported diagnosis of chronic diseases, including hypertension, diabetes, cancer, chronic lung disease, heart disease, stroke, and kidney disease. Continuous measurements of SBP and DBP were also adjusted in all analyses in the current study, to show the magnitude of associations between anti-hypertensive medication and cognitive aging independently from BP levels. Physical disability was defined as difficulties in one or more of the following basic activities of daily living: bathing, dressing, eating, getting in/out of bed, and using the toilet.

### Statistical analysis

The mean (SD) or the median (interquartile range [IQR]) were used for descriptive statistics of continuous variables and numbers (percentage) for categorical variables. Differences in characteristics were tested using the analysis of variance, chi-square test, or Kruskal–Wallis test.

To assess longitudinal associations between anti-hypertensive medication and the rate of cognitive aging, linear mixed models were applied to analyze the repeated cognitive measurement data. Such models have been widely embraced for handling longitudinal measurements of continuous outcomes and can incorporate all available follow-up data to derive the rate of change in certain outcomes [5, 24, 25]. Following terms were included in models as fixed effects, including main effects of anti-hypertensive medication, time (follow-up year from baseline, as a continuous variable), anti-hypertensive medication × time interaction, and covariates. The coefficient of the anti-hypertensive medication × time interaction represents longitudinal associations between anti-hypertensive medication use and the annual rate of cognitive decline (SD/year), with a positive value indicating decelerated decline and a negative value indicating accelerated decline, respectively. At the participant level, we also fitted random intercept and random slope of time, to account for the individual difference in baseline cognitive performance and longitudinal rate of cognitive aging during follow-up, respectively [26].

To examine the interaction between SES and anti-hypertensive medication, we performed the interaction analysis. Aligning with a previous study [26], we fitted linear mixed models to test for the interaction, by adding fixed effects of the latent SES variable, two-way interactions of anti-hypertensive medication with latent SES variable and time, and three-way interactions between anti-hypertensive medication, latent SES variable, and time. We also evaluated joint associations between anti-hypertensive medication, SES indicators, and cognitive aging rate. Joint categories were created by combining anti-hypertensive medication use and SES variables. Then, we replaced the anti-hypertensive medication variable with the joint categories in linear mixed models and re-assessed the associations with cognitive aging.

Several sensitivity analyses were conducted. First, as the anti-hypertensive medication adherence assessed during waves 1 and 2 overlapped with the cognitive measurements during waves 1 and 2, we excluded individuals with reported memory-related disease or developed cognitive impairment during waves 1 and 2, to further address the reverse causation challenge. Based on previous studies [5, 27], we defined cognitive impairment as a global cognitive Z score below −1.5, as compared to the population's average cognitive performance. Then, we re-evaluated the associations between anti-hypertensive medication and cognitive aging. Second, participants in the CHARLS were also asked about the usage of Chinese traditional medicine to treat hypertension, which was further considered when evaluating anti-hypertensive medication. Third, in addition to global cognition and memory, we evaluated associations between anti-hypertensive medication and cognitive aging in other cognitive domains, including executive function and orientation. Fourth, in addition to baseline BP measurements adjusted in primary analysis, follow-up BP measurements (in wave 2) were further adjusted. Fifth, to test whether associations between anti-hypertensive medication and cognitive aging were independent of SES and examine the mediation effect of SES, we repeated linear mixed model analysis by simultaneously controlling for overall SES and SES indicators. Likewise, we conducted linear mixed model analysis to examine associations between overall SES and longitudinal cognitive aging, with and without controlling for anti-hypertensive medication. Sixth, to help evaluate the reverse causation challenge, we analyzed associations between baseline and follow-up cognitive performance with the longitudinal adherence to anti-hypertensive medication, restricted to individuals with hypertension. Seventh, we incorporated data from waves 1 to 4 to evaluate the long-term adherence to anti-hypertensive medication, by summing up the number of waves reporting using anti-hypertensive medication. Then we examined associations between the updated longitudinal adherence score and cognitive aging rate. Eighth, aligning with our previous work [19], we further applied an inverse probability weighting (IPW) approach to address the potential selection bias, as a large number of participants were excluded in the current study. The IPW approach was proposed to re-weight the included study samples, while the analytical weight for each individual was calculated as the inverse of the probability of being included in the analysis [28]. Binary logistic regression was used to estimate the weights, with major baseline characteristics included as explanatory variables. Absolute standardized mean differences were used for assessing the difference between included and excluded participants [29]. We conducted a non-response analysis to compare baseline characteristics of included and excluded participants, to help evaluate the selection bias [19]. Finally, we explored joint associations between anti-hypertensive medication use and baseline age, by categorizing participants into age groups by 60 (aged < 60 years and aged ≥ 60 years) and 65 (aged < 65 years and aged ≥ 65 years), respectively.

Statistical analysis was conducted using SAS version 9.4 (SAS Institute) and R language 4.3.1 (R Foundation, Vienna, Austria), with a two-tailed alpha of 0.05 considered statistically significant.

## Results

### Study population

Among the included 9229 participants (mean [SD] age: 57.1 [8.9] years; men: 50.8%), 5631 participants were categorized as normotension, 1537 participants were using anti-hypertensive medication at baseline, and 2061 participants not taking anti-hypertensive medication at baseline. As shown in Table 1, among 3598 participants with hypertension, those taking anti-hypertensive medication were more likely to be women, had better overall SES, less prevalent alcohol consumption and smoking, lower BP levels, and more prevalent major chronic conditions (except for cancer, lung disease, and kidney disease) than those not taking medication at baseline (all *P* < 0.05 for comparison). As shown in Additional file 1: Table S3, among 3598 participants with hypertension, 1588 participants had low adherence, 858 participants had moderate adherence, and 1152 participants had high adherence to anti-hypertensive medication, respectively. Similar comparisons were observed between those with better anti-hypertensive medication adherence and low adherence, shown in Additional file 1: Table S3 (all *P* < 0.05 for comparison). As shown in Fig. 1B, the proportion of anti-hypertensive medication use at wave 1 and wave 2 was 42.7% and 45.2%, respectively. A statistically significant elevation of 2.5% (95% CI, 0.2% to 4.7%; *P* = 0.036) was observed in proportion using medication (Fig. 1B).

**Table 1:** Baseline characteristics of participants by baseline anti-hypertensive medication use

Characteristics	Participants, No. (%)				*P* value^a^
	**All participants** ***N* = 9229**	**With hypertension**		**Normotension** ***N* = 5631**	
		**Not using medication** ***N* = 2061**	**Using medication** ***N* = 1537**		
Age, mean (SD), y	57.1 (8.9)	59.1 (9.3)	60.2 (8.5)	55.4 (8.4)	< 0.001
Sex					< 0.001
Men	4687 (50.8)	1106 (53.7)	719 (46.8)	2862 (50.8)	
Women	4542 (49.2)	955 (46.3)	818 (53.2)	2769 (49.2)	
Overall socioeconomic status^b^					0.003
Lower	8375 (90.7)	1910 (92.7)	1390 (90.4)	5075 (90.1)	
Upper	854 (9.3)	151 (7.3)	147 (9.6)	556 (9.9)	
Education					0.007
Less than high school	8066 (87.4)	1846 (89.6)	1344 (87.4)	4876 (86.6)	
High school or equivalent	1020 (11.1)	182 (8.8)	171 (11.1)	667 (11.8)	
College and higher	143 (1.5)	33 (1.6)	22 (1.4)	88 (1.6)	
Annual family income^c^					< 0.001
Below the median	4614 (50.0)	1113 (54.0)	776 (50.5)	2725 (48.4)	
Above the median	4615 (50.0)	948 (46.0)	761 (49.5)	2906 (51.6)	
Medical insurance coverage					0.06
Uninsured	575 (6.2)	149 (7.2)	83 (5.4)	343 (6.1)	
Insured	8654 (93.8)	1912 (92.8)	1454 (94.6)	5288 (93.9)	
Employment status					0.001
Unemployed	269 (2.9)	53 (2.6)	68 (4.4)	148 (2.6)	
Employed	8960 (97.1)	2008 (97.4)	1469 (95.6)	5483 (97.4)	
Living alone	881 (9.5)	259 (12.6)	189 (12.3)	433 (7.7)	< 0.001
Physical exercise	2721 (29.5)	567 (27.5)	387 (25.2)	1767 (31.4)	< 0.001
Alcohol consumption	1623 (17.6)	427 (20.7)	204 (13.3)	992 (17.6)	< 0.001
Current smoking	3004 (32.5)	741 (36.0)	384 (25.0)	1879 (33.4)	< 0.001
Systolic blood pressure, mean (SD), mmHg	128.4 (20.8)	147.8 (18.4)	143.0 (21.7)	117.3 (11.6)	< 0.001
Diastolic blood pressure, mean (SD), mmHg	75.5 (12.1)	85.0 (11.8)	81.6 (12.4)	70.4 (8.9)	< 0.001
Physical disability	1046 (11.3)	244 (11.8)	243 (15.8)	559 (9.9)	< 0.001
Hypertension	3598 (39.0)	2061 (100.0)	1537 (100.0)	0 (0.0)	< 0.001
Diabetes	564 (6.1)	126 (6.1)	232 (15.1)	206 (3.7)	< 0.001
Cancer	78 (0.8)	18 (0.9)	16 (1.0)	44 (0.8)	0.60
Chronic lung disease	858 (9.3)	214 (10.4)	156 (10.1)	488 (8.7)	0.03
Heart disease	1071 (11.6)	225 (10.9)	402 (26.2)	444 (7.9)	< 0.001
Stroke	189 (2.0)	41 (2.0)	84 (5.5)	64 (1.1)	< 0.001
Kidney disease	496 (5.4)	121 (5.9)	92 (6.0)	283 (5.0)	0.17
Memory function score, mean (SD)	7.6 (3.3)	7.4 (3.3)	7.4 (3.0)	7.7 (3.3)	< 0.001
Executive function score, median (IQR)	4.0 (3.0–5.0)	4.0 (3.0–5.0)	4.0 (3.0–5.0)	4.0 (3.0–5.0)	0.02
Orientation score, median (IQR)	3.0 (3.0–4.0)	3.0 (3.0–4.0)	4.0 (3.0–4.0)	3.0 (3.0–4.0)	< 0.001

*Abbreviations: SD* Standard deviation, *IQR* Interquartile range

^a^*P* value reported for differences between groups using analysis of variance, Kruskal–Wallis test, or chi-squared test

^b^Latent class analysis was used for classifying the overall socioeconomic status, derived using indicators of education, income, employment status, and medical insurance

^c^Median of annual family income was $2877.7 (to convert to Chinese yuan, multiplied by 6.3)

### Associations between anti-hypertensive medication and cognitive aging

As shown in Table 2, compared to participants not using anti-hypertensive medication, those taking anti-hypertensive medication at baseline had a significantly decelerated decline in global cognition (β = 0.014; 95% confidence interval [CI], 0.003 to 0.025 SD/year; *P* = 0.01) and memory function (β = 0.021; 95% CI, 0.008 to 0.034 SD/year; *P* = 0.001), respectively. Similar decelerated cognitive decline was also observed in the normotension group, shown in global cognition (β = 0.018; 95% CI, 0.009 to 0.026 SD/year; *P* < 0.001) and memory function (β = 0.028; 95% CI, 0.018 to 0.038 SD/year; *P* < 0.001), respectively (Table 2). Compared to the normotension, participants not using anti-hypertensive medication at baseline had significantly accelerated decline in global cognition (β = −0.018; 95% CI, −0.026 to −0.009 SD/year; *P* < 0.001) and memory function (β = −0.028; 95% CI, −0.038 to −0.018 SD/year; *P* < 0.001), respectively, while no significant differences were observed for those taking medication at baseline (Table 2).

**Table 2:** Associations between anti-hypertensive medication use and annual rate of change in cognitive function

Anti-hypertensive medication	No. of participants	Global cognition (SD/y)		Memory function (SD/y)	
		**β (95% CI)**^**a**^	***P* value**	**β (95% CI)**^**a**^	***P* value**
Baseline anti-hypertensive medication use					
Hypertension not using medication	2061	0 [Reference]	NA	0 [Reference]	NA
Hypertension using medication	1537	0.014 (0.003 to 0.025)	0.01	0.021 (0.008 to 0.034)	0.001
Normotension	5631	0.018 (0.009 to 0.026)	< 0.001	0.028 (0.018 to 0.038)	< 0.001
Compared to normotension					
Normotension	5631	0 [Reference]	NA	0 [Reference]	NA
Hypertension not using medication	2061	−0.018 (−0.026 to −0.009)	< 0.001	−0.028 (−0.038 to −0.018)	< 0.001
Hypertension using medication	1537	−0.004 (−0.013 to 0.006)	0.44	−0.007 (−0.018 to 0.004)	0.23
Longitudinal adherence to anti-hypertensive medication					
Hypertension, low adherence	1588	0 [Reference]	NA	0 [Reference]	NA
Hypertension, moderate adherence	858	0.012 (−0.002 to 0.026)	0.09	0.006 (−0.010 to 0.023)	0.45
Hypertension, high adherence	1152	0.014 (0.002 to 0.027)	0.02	0.023 (0.008 to 0.038)	0.003
Normotension	5631	0.019 (0.010 to 0.028)	0.001	0.028 (0.017 to 0.039)	< 0.001
Compared to normotension					
Normotension	5631	0 [Reference]	NA	0 [Reference]	NA
Hypertension, low adherence	1588	−0.019 (−0.028 to −0.010)	< 0.001	−0.028 (−0.039 to −0.017)	< 0.001
Hypertension, moderate adherence	858	−0.007 (−0.019 to 0.005)	0.23	−0.022 (−0.036 to −0.008)	0.003
Hypertension, high adherence	1152	−0.005 (−0.015 to 0.006)	0.37	−0.005 (−0.017 to 0.008)	0.45

Adjusted covariates included age, sex, education, cohabitation status, physical activity, alcohol consumption, current smoking, physical disability, hypertension, diabetes, cancer, chronic lung disease, heart disease, stroke, kidney disease, and baseline measurements of blood pressure (systolic and diastolic blood pressure)

*NA* Not applicable

^a^β coefficient was estimated using linear mixed models, with a positive value representing decelerated cognitive decline in comparison

For the longitudinal adherence to anti-hypertensive medication, shown in Table 2, those with either high adherence or normotension had a significantly decelerated decline in both global cognition and memory function, compared to the low adherence group. Similarly, when compared to the normotension group, those with low medication adherence had significantly accelerated decline in global cognition (β = −0.019; 95% CI, −0.028 to −0.010 SD/year; *P* < 0.001) and memory function (β = −0.028; 95% CI, −0.039 to −0.017 SD/year; *P* < 0.001), while no significant differences were observed for those with high medication adherence (Table 2).

**Fig. 2:**
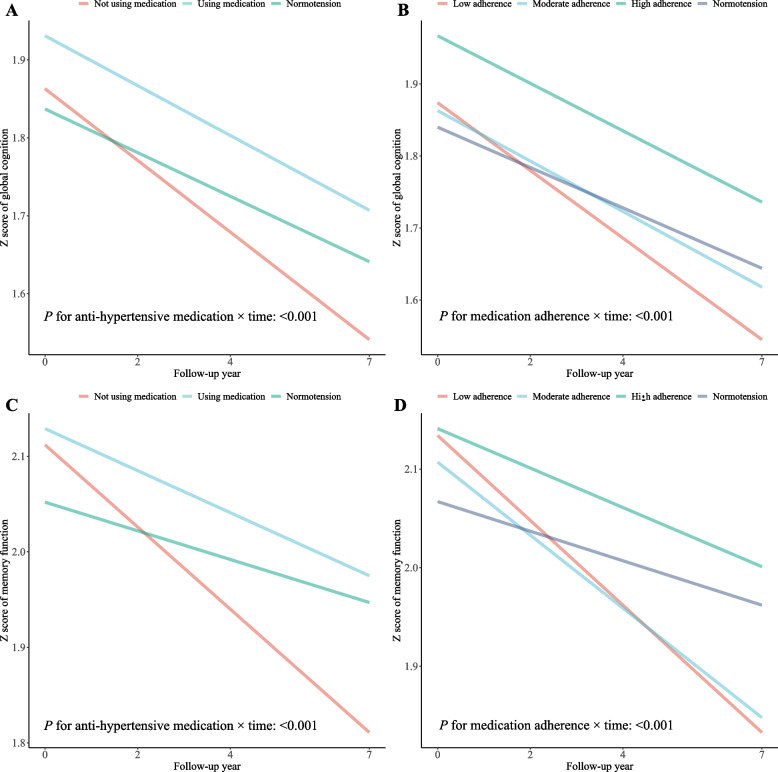
Estimated cognitive trajectories by anti-hypertensive medication use. **A** Estimated trajectories of global cognition according to baseline anti-hypertensive medication use. **B** Estimated trajectories of global cognition according to longitudinal anti-hypertensive medication adherence. **C** Estimated trajectories of memory function according to baseline anti-hypertensive medication use. **D** Estimated trajectories of memory function according to longitudinal anti-hypertensive medication adherence. Linear mixed models in Table 2 were applied to estimate the cognitive trajectories, with identical covariates adjusted

Cognitive trajectories by anti-hypertensive medication use were presented in Fig. 2, which also showed steeper cognitive declines in participants not using anti-hypertensive medication at baseline or with low adherence during follow-up, compared to their counterparts using medication or with high adherence (all *P* < 0.001 for medication × time interaction).

### Interactions and joint associations between anti-hypertensive medication, socioeconomic status, and cognitive aging

**Fig. 3:**
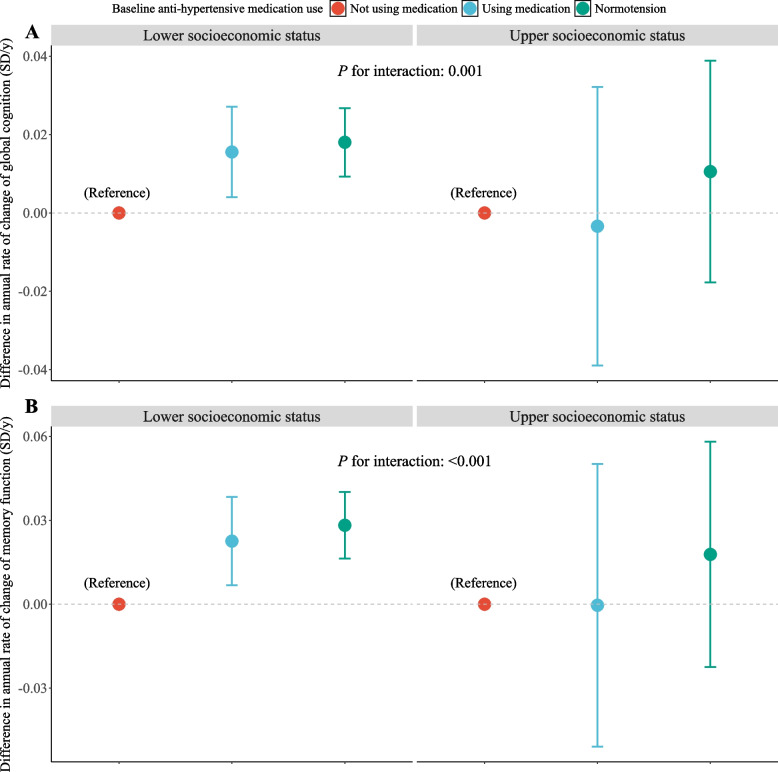
Interactions between baseline anti-hypertensive medication use and overall socioeconomic status with cognitive decline rate. **A** Interactions between baseline anti-hypertensive medication use and overall socioeconomic status with declines in global cognition; (**B**) Interactions between baseline anti-hypertensive medication use and overall socioeconomic status with declines in memory function. Differences in the annual rate of cognitive change were estimated using linear mixed models, controlling for age, sex, cohabitation status, physical activity, alcohol consumption, current smoking, physical disability, hypertension, diabetes, cancer, chronic lung disease, heart disease, stroke, kidney disease, and baseline measurements of blood pressure (systolic and diastolic blood pressure)

**Fig. 4:**
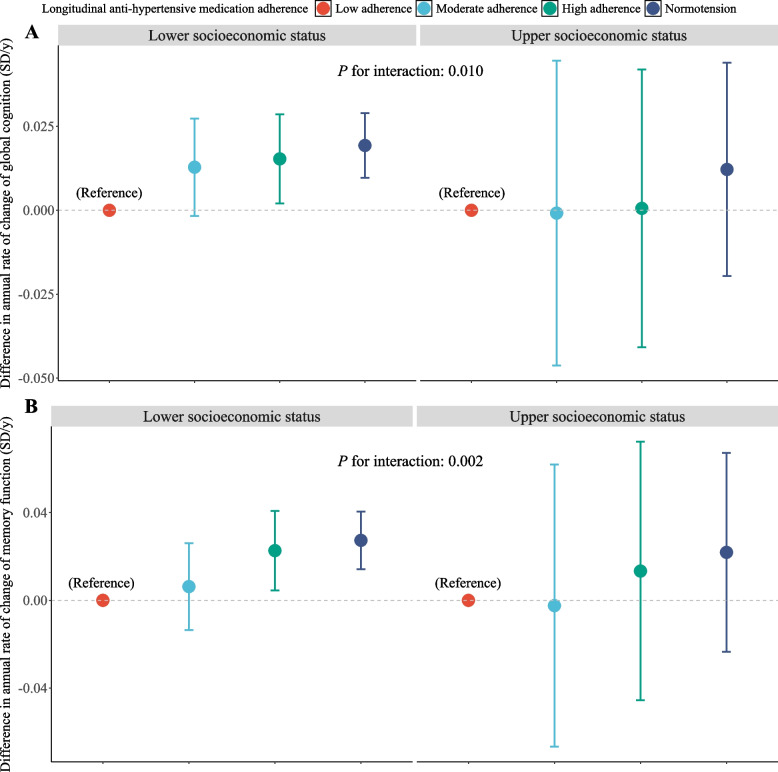
Interactions between longitudinal anti-hypertensive medication adherence and overall socioeconomic status with cognitive decline rate. **A** Interactions between longitudinal anti-hypertensive medication adherence and overall socioeconomic status with declines in global cognition; (**B**) Interactions between longitudinal anti-hypertensive medication adherence and overall socioeconomic status with declines in memory function. Differences in the annual rate of cognitive change were estimated using linear mixed models, controlling for age, sex, cohabitation status, physical activity, alcohol consumption, current smoking, physical disability, hypertension, diabetes, cancer, chronic lung disease, heart disease, stroke, kidney disease, and baseline measurements of blood pressure (systolic and diastolic blood pressure)

As shown in Figs. 3 and 4, significant interactions were observed between anti-hypertensive medication and overall SES. As presented in Fig. 3A and B, using anti-hypertensive medication was consistently associated with slower decline rates in global cognition and memory in the lower SES subgroup. By contrast, in the upper SES subgroup, no significant differences were observed in cognitive decline rates between participants using and not using medication, with significant interactions observed (all *P* for interaction < 0.05). Likewise, as shown in Fig. 4A and B, the high longitudinal adherence to anti-hypertensive medication was consistently associated with slower cognitive aging rates in the lower SES subgroup, compared to the null associations in the upper SES subgroup. Significant interactions were observed (all *P* for interaction < 0.05).

Compared to participants with low income and not using medication, those with low income and using medication had significantly decelerated global cognitive decline (β = 0.022; 95% CI, 0.007 to 0.038 SD/year; Additional file 1: Fig. S2). Compared to participants with low education and not using medication, those with low education and using medication had significantly decelerated global cognitive decline (β = 0.015; 95% CI, 0.004 to 0.027 SD/year; Additional file 1: Fig. S2). No significant joint associations were observed for employment status and medical insurance coverage (Additional file 1: Fig. S2). Similar patterns of associations were observed in memory decline, showing significant joint associations between baseline anti-hypertensive medication, income, and education (Additional file 1: Fig. S3).

Similarly, as presented in Additional file 1: Fig. S4, for participants with low income or education, significant decelerated global cognitive decline was only observed in those with high anti-hypertensive medication adherence or had normotension. Compared to participants with low income and low adherence, shown in Additional file 1: Fig. S4, those with low income and high adherence had significantly decelerated global cognitive decline (β = 0.026; 95% CI, 0.008 to 0.043 SD/year). Similarly, compared to participants with low education and low adherence, those with low education and high adherence had significantly decelerated global cognitive decline (β = 0.015; 95% CI, 0.001 to 0.028 SD/year; Additional file 1: Fig. S4). No significant joint associations were observed for employment status and medical insurance coverage (Additional file 1: Fig. S4). Similar patterns of associations were also observed for memory decline, shown in Additional file 1: Fig. S5.

### Sensitivity analyses

The observed associations were not materially changed after addressing reverse causation (Additional file 1: Table S4) and accounting for using Chinese traditional medicine to treat hypertension (Additional file 1: Table S5). For the decline in executive function and orientation (Additional file 1: Table S6), most associations between anti-hypertensive medication use and cognitive aging did not reach statistical significance. Further controlling for BP measurements during follow-up also did not change the results (Additional file 1: Table S7). Controlling for the overall SES variable and SES indicators did not attenuate associations between anti-hypertensive medication and cognitive aging, shown in Additional file 1: Table S8 and Additional file 1: Table S9. The upper SES was consistently associated with slower annual decline rates in global cognition and memory, no matter controlling for anti-hypertensive medication or not (Additional file 1: Table S10). Further analysis showed that both the better baseline cognitive performance and the improved cognition during follow-up were associated with elevated odds of high adherence to anti-hypertensive medication, shown in Additional file 1: Table S11. As shown in Additional file 1: Table S12, compared to hypertension participants reported 0 wave of using medication, hypertension participants reported all 4 waves of medication use had significantly decelerated memory decline (β = 0.020; 95% CI, 0.002 to 0.038 SD/year), while no significant difference was observed in global cognitive decline (β = 0.014; 95% CI, −0.001 to 0.030 SD/year). Similarly, participants reported medication use for 3 or 4 waves did not have accelerated declines in global cognition or memory, compared to the normotension group (Additional file 1: Table S12).

As shown in Additional file 1: Fig. S6, after the IPW procedure, all baseline characteristics were well-balanced between included and excluded participants. Then, based on the IPW-weighted samples, all primary results were not materially altered, showing consistent associations between anti-hypertensive medication use and decelerated cognitive decline (Additional file 1: Table S13). A total of 8479 participants were excluded from the analysis, who were generally older, more likely to be women, less educated, and living alone, compared to included participants (Additional file 1: Table S14). Using anti-hypertensive medication at baseline was not associated with decelerated aging in global cognition in participants aged ≥ 60 or 65 years (Additional file 1: Fig. S7), while significantly decelerated memory decline was observed in medication users of the same age groups (Additional file 1: Fig. S7). Similar joint association patterns were observed between anti-hypertensive medication adherence and baseline age (Additional file 1: Fig. S8), showing significantly decelerated memory decline associated with high medication adherence in participants aged ≥ 60 or 65 years.

## Discussion

In the 7-year longitudinal cohort of Chinese middle-aged and older community-dwelling adults ≥ 45 years, we found both using anti-hypertensive medication at baseline and high adherence during follow-up, compared to not using medication at baseline and low adherence during follow-up, were associated with a slower rate of cognitive aging, independently from BP levels and other factors. Intriguingly, participants taking medication at baseline or had high adherence during follow-up, compared to those with normotension, did not have accelerated cognitive aging. The observed associations remained robust to sensitivity analyses, including the analysis to address reverse causation and controlling for the SES. Moreover, significant interactions and joint associations were observed between anti-hypertensive medication and SES in cognitive aging, with more pronounced associations observed in the lower SES subgroup, supporting the potential significance of promoting anti-hypertensive medication use in addressing the ever-expanding socioeconomic inequalities in healthy cognitive aging. To the best of our current knowledge, this is the first longitudinal cohort study to simultaneously evaluate prospective associations of anti-hypertensive medication use and SES with long-term cognitive aging in Chinese middle-aged and older adults, with robust findings observed.

Previous studies have explored associations between anti-hypertensive medication use or BP control in relations to cognitive outcomes, reporting generally similar findings with ours. The SPRINT trial confirmed that intensive SBP control could reduce the risk of mild cognitive impairment by 19% among adults aged 50 years or older with hypertension but without diabetes or a history of stroke [6]. Although the SPRINT did not observe a significant reduced risk of probable dementia, it supported the cognitive benefits of controlling SBP. Another meta-analysis of five randomized trials showed the efficacy of antihypertensive treatment use in reducing dementia risk by 13% in patients aged > 65 years [7]. There were also observational reports regarding associations between anti-hypertensive medication and cognitive outcomes. A meta-analysis of six prospective cohort studies found in those with SBP/DBP ≥ 140/90 mm Hg, using anti-hypertensive medication was associated with a 12% reduced dementia risk, while no significant associations were found in the normal BP stratum [8]. Another review investigated associations of anti-hypertensive medication classes with incident cognitive decline, showing no significant associations both in individuals aged > 65 years and ≤ 65 years, possibly owing to the limited data of participants aged ≤ 65 years [9]. So far, limited studies have investigated the role of anti-hypertensive medication in the longitudinal cognitive aging process in hypertension participants, especially in a relatively young population. And our study, with generally similar findings observed, additionally found that adhering to anti-hypertensive medication was associated with a slower longitudinal cognitive decline rate in middle-aged and older hypertension individuals aged ≥ 45 years. Moreover, further interaction analysis showed that observed associations were more pronounced in individuals with low SES, which have rarely been reported by previous investigations. A recent study examined associations between untreated and treated blood pressure levels and longitudinal cognitive decline [12]. Unlike our study, the study primarily focused on BP levels, with anti-hypertensive medication status analyzed as a potential effect modifier [12]. Despite the distinctive research objectives, the authors found that anti-hypertensive medication use attenuated associations between elevated BP levels and accelerated cognitive decline [12], which aligned with our findings by supporting the potential protective role of anti-hypertensive medication against cognitive aging.

Our study adds to the current literature mainly by showing that both baseline and longitudinal anti-hypertensive medication use were associated with a slower cognitive aging rate, independently from BP status and other characteristics. More importantly, we found that participants adhering to anti-hypertensive medication did not suffer accelerated cognitive aging, compared to their counterparts with normotension. On the contrary, those not taking anti-hypertensive medication or with poor medication adherence, consistently had accelerated cognitive decline than those with normotension. The finding is intriguing, showing that adhering to anti-hypertensive medication could be beneficial in preventing long-term cognitive aging, which might help to ease the severe dementia-related burden in the hypertension population. However, we found that better cognition was associated with higher adherence to anti-hypertensive medication. Previous studies also reported associations between worse cognitive performance and poor adherence to anti-hypertensive medication [30, 31]. Therefore, it is possible that our findings represent the potential bias of reverse causality and should be interpreted cautiously. Although we have performed a sensitivity analysis by excluding participants with memory disorders or cognitive impairment, the bias cannot be entirely eliminated. Further investigations capable of addressing the reverse causality bias are warranted to validate our findings.

Another intriguing finding of our study was the significant interaction between anti-hypertensive medication and SES in associations with cognitive aging. As an important social determinant of health, disadvantaged SES has been found to be directly associated with acceleration of aging in multiple facets, including accelerated cognitive aging [14]. Moreover, SES disparities impact the access to health care services, highlighting its role in determining health and healthcare needs of older adults [32]. It has also been found the higher SES was related with the better adherence to anti-hypertensive medication [16]. Hence, it is of crucial significance to fully understand the role of SES in associations between anti-hypertensive medication adherence and cognitive aging, providing enriched insights to address ever-expanding SES-related health disparities and achieve inclusive healthy aging.

We found adhering to anti-hypertensive medication was more evidently associated with a slower cognitive aging rate in participants with low SES, compared to their high SES counterparts. Moreover, we found significant joint associations between anti-hypertensive medication and single SES indicators in cognitive aging rate, mainly for education and income. Using anti-hypertensive medication was consistently associated with decelerated cognitive aging in hypertension participants with either low income or educational level. Collectively, these findings are also of great public health implications. Disparities in SES have been associated with health inequalities for decades, especially in inequalities related to the healthy aging process. For instance, SES has been found to impact cognitive ability among Chinese older adults and those with higher SES consistently had better cognitive performance [33]. Another cohort study found low SES individuals, compared to high SES counterparts, had a 340% higher risk of early-onset dementia and a 90% higher risk of late-onset dementia, respectively [15]. Likewise, low wealth was also found to account for a 68% higher dementia risk in people aged 65 years and older [34]. Moreover, individuals with better SES could also get ahead in terms of timely diagnosis. In a register-based cross-sectional study in Denmark, people with the upper tertile of household income, compared to the lower-tertile, not only had lower odds of dementia diagnosis after referral, but had less severe cognitive stage at diagnosis, indicating a better chance of receiving an earlier dementia diagnosis [35]. Consequently, high SES individuals could gain various advantages in healthy cognitive aging, while those with disadvantaged SES would confront with more severe dementia-related burden. Our findings support the huge significance of promoting anti-hypertensive medication in low SES individuals living with hypertension to prevent cognitive deterioration at the early preclinical stage, especially in those with low income or education. Further investigations are warranted to confirm such findings.

There are other notable findings of the study. First, associations between anti-hypertensive medication and cognitive aging were more evident and consistent for the memory function, compared to other cognitive domains. Our previous work revealed that long-term BP exposure was more evidently associated with accelerated memory decline [5]. Considering that accelerated memory decline is one of the shared and early indicators of dementia onset [5], our findings highlight the potential clinical relevance of adhering to anti-hypertensive medication for timely dementia prevention. In addition, we found among older adults aged ≥ 60 or 65 years, using anti-hypertensive medication was consistently associated with a decelerated decline in memory, which also supports the initiation of anti-hypertensive medication among older individuals with hypertension. Second, in addition to statistical significance, the current study also provided findings of potential clinical significance. For instance, compared to low medication adherence participants, high adherence participants on average had a decelerated memory decline of 0.023 (95% CI, 0.008 to 0.038) SD/year. According to a previous review, a change of 0.5 SD in the cognitive score can be viewed as clinically important [36]. That is saying, taking the upper bound of difference, e.g. 0.038 SD/year in memory decline rate, as an example, individuals with high anti-hypertensive medication adherence would have clinically meaningful prevented memory deterioration in almost 13 years (0.5/0.038), compared to their low adherence counterparts. Acknowledgment of such implications could help clinicians to improve practice, not just in hypertension management, but in early prevention of cognitive deterioration among aging individuals. Third, the observed associations remained consistent even after controlling for follow-up BP measurements, indicating other potential explanations behind the potential neurocognitive benefits of anti-hypertensive medication, beyond longitudinal BP control pattern.

Our study has several strengths. First, based on a large national survey with longitudinal repeated measurements of cognitive function, we were able to evaluate the annual cognitive aging rate as the primary outcome. As one of the most important early indications of dementia onset [10], evaluating associations between anti-hypertensive medication and accelerated cognitive decline provides important implications for practice, supporting the potential significance of anti-hypertensive medication in preventing early cognitive deterioration at the pre-clinical phase of dementia. Second, unlike previous investigations, the current study, based on longitudinal data, also evaluated the long-term adherence to anti-hypertensive medication during follow-up, accounting for changes in anti-hypertensive medication use. We found that hypertension participants persistently adhering to medication during follow-up presented a significantly slower cognitive aging rate than counterparts with low adherence, which supported the importance of monitoring and promoting anti-hypertensive medication adherence in the long-term. Third, our study was based on the general community-dwelling middle-aged and older adults in China. With the unique population, we were able to examine the role of adhering to anti-hypertensive medication against accelerated cognitive aging in a setting reflecting the real-world practice of hypertension management. Intriguingly, we found the overall proportion adhering to anti-hypertensive medication was relatively low, only 32% of patients persistently adhered to medication during follow-up. This was in line with a recent study based on health care utilization databases of Lombardy, Italy, with only 34% patients reporting anti-hypertensive medication use in more than 75% of the follow-up [11]. Hence, our findings provide more enriched insights for the current practice by highlighting the challenge of low anti-hypertensive treatment adherence in general settings. Fourth, the potential role of SES was comprehensively investigated in our study. Based on the LCA approach, we constructed an overall SES index by effectively incorporating multiple SES indicators. Then we examined the interplay between anti-hypertensive medication and overall SES, observing more evident associations in low SES participants. We also evaluated joint associations between anti-hypertensive medication and single SES indicators. We found that individuals of low income or education were more likely to benefit from high adherence to anti-hypertensive medication. These findings provide novel insights for addressing ever-expanding SES disparities in cognitive aging. Finally, our findings are relevant for the current practice, providing implications from both clinical and public health perspectives. Several sensitivity analyses were also conducted to address potential biases, including the efforts addressing reverse causality and selection bias, with robust findings observed.

There also exist important limitations. First, self-reported data was collected for categorizing anti-hypertensive medication status, leading to potential misclassification bias. We also could not differentiate between anti-hypertensive medication classes, owing to data restrictions. Second, the administered cognitive batteries only involved three cognitive domains, preventing us from capturing cognitive aging in other cognitive domains. Third, the challenge of reverse causation persisted. Although we conducted a sensitivity analysis by excluding cognitively impaired individuals during medication assessment, the reverse causation challenge could not be eliminated. Cautions are therefore warranted to appropriately understand and interpret our findings. Fourth, a considerable number of participants were excluded, resulting in potential selection bias. Despite the IPW analysis yielding similar findings, we could not entirely preclude the selection bias. Finally, due to the nature of observational studies, we could not eliminate the impact of residual confounding, which impedes further steps toward conclusively defining causal relationships [37]. Moreover, due to data restrictions, we could not identify possible factors fully explaining observed associations between anti-hypertensive medication and longitudinal cognitive aging. Further investigations are therefore needed to explore potential operating mechanisms.

## Conclusions

We found that adhering to anti-hypertensive medication was associated with decelerated cognitive aging in Chinese middle-aged and older community-dwelling adults ≥ 45 years, especially in participants with low SES. No significant differences were observed in cognitive aging rates between hypertension participants adhering to anti-hypertensive medication and counterparts with normotension. These findings indicate that promoting anti-hypertensive medication use could be important to achieve healthy and inclusive cognitive aging in general Chinese middle-aged and older adults living with hypertension.

## Supplementary Information

Additional file 1: Supplemental Methods. Details of cognitive function assessment in the CHARLS. Table S1. Item-response probabilities for the latent class analysis for classifying socioeconomic status. Table S2. Comparison of socioeconomic status indicators by identified latent classes. Table S3. Baseline characteristics of participants by longitudinal adherence to anti-hypertensive medication. Table S4. Associations between anti-hypertensive medication use and the annual rate of change in cognitive function, further excluding individuals with reported memory-related disease or developed cognitive impairment during wave 1 and wave 2. Table S5. Associations between anti-hypertensive medication use and the annual rate of change in cognitive function, further accounting for the use of Chinese traditional medicine. Table S6. Associations between anti-hypertensive medication use and annual rate of change in cognitive function, accounting for other cognitive domains. Table S7. Associations between anti-hypertensive medication use and annual rate of change in cognitive function, further controlling for blood pressure follow-up measurements. Table S8. Associations between anti-hypertensive medication use and the annual rate of change in cognitive function, controlling for the overall socioeconomic status. Table S9. Associations between anti-hypertensive medication use and the annual rate of change in cognitive function, controlling for all indicators of socioeconomic status. Table S10. Associations between overall socioeconomic status and the annual rate of change in cognitive function, controlling for anti-hypertensive medication use. Table S11. Associations between baseline cognition and follow-up cognitive changes with the adherence to anti-hypertensive medication in participants with hypertension. Table S12. Associations between longitudinal anti-hypertensive medication adherence and the annual rate of change in cognitive function, incorporating data from wave 1 to wave 4. Table S13. Associations between anti-hypertensive medication use and the annual rate of change in cognitive function, based on inverse probability-weighted samples. Table S14. Baseline characteristics comparison between participants included and excluded from analysis in CHARLS. Figure S1. Participants selection diagram. Figure S2. Joint associations of baseline anti-hypertensive medication use and socioeconomic status with declines in global cognition. Figure S3. Joint associations of baseline anti-hypertensive medication use and socioeconomic status with declines in memory function. Figure S4. Joint associations of longitudinal anti-hypertensive medication adherence and socioeconomic status with declines in global cognition. Figure S5. Joint associations of longitudinal anti-hypertensive medication adherence and socioeconomic status with declines in memory function. Figure S6. Love plot assessing the difference in baseline characteristics between participants included in and excluded from the primary analysis, before and after inverse probability weighting. Figure S7. Joint associations of baseline anti-hypertensive medication use and baseline age with cognitive aging. Figure S8. Joint associations of longitudinal anti-hypertensive medication adherence and baseline age with cognitive aging.

## Data Availability

Original survey datasets from the CHARLS are freely available to all bonafide researchers. Access to data can be obtained by visiting their official websites (http://charls.pku.edu.cn/en/).

## Abbreviations

SES: Socioeconomic status
CHARLS: China Health and Retirement Longitudinal Study
SBP: Systolic blood pressure
DBP: Diastolic blood pressure
CI: Confidence interval
SD: Standard deviation

## References

[1] Livingston G, Huntley J, Sommerlad A, Ames D, Ballard C, Banerjee S, et al. Dementia prevention, intervention, and care: 2020 report of the Lancet Commission. Lancet. 2020;396:413–46. 32738937 10.1016/S0140-6736(20)30367-6 PMC7392084

[2] Alzheimer’s Disease International. World Alzheimer Report 2023 Reducing dementia risk: never too early, never too late. 2023. https://www.alzint.org/resource/world-alzheimer-report-2023/. Accessed 25 Jun 2024.

[3] World Health Organization. Global action plan on the public health response to dementia 2017 - 2025. 2017. https://www.who.int/publications/i/item/global-action-plan-on-the-public-health-response-to-dementia-2017---2025. Accessed 4 Dec 2023.

[4] Mukadam N, Sommerlad A, Huntley J, Livingston G. Population attributable fractions for risk factors for dementia in low-income and middle-income countries: an analysis using cross-sectional survey data. Lancet Glob Heal. 2019;7:e596-603. 10.1016/S2214-109X(19)30074-9 PMC7617123 31000129

[5] Li C, Zhu Y, Ma Y, Hua R, Zhong B, Xie W. Association of Cumulative Blood Pressure With Cognitive Decline, Dementia, and Mortality. J Am Coll Cardiol. 2022;79:1321–35. 35393012 10.1016/j.jacc.2022.01.045

[6] Williamson JD, Pajewski NM, Auchus AP, Bryan RN, Chelune G, Cheung AK, et al. Effect of Intensive vs Standard Blood Pressure Control on Probable Dementia: A Randomized Clinical Trial. JAMA. 2019;321:553–61. 30688979 10.1001/jama.2018.21442 PMC6439590

[7] Peters R, Xu Y, Fitzgerald O, Aung HL, Beckett N, Bulpitt C, et al. Blood pressure lowering and prevention of dementia: an individual patient data meta-analysis. Eur Heart J. 2022. 10.1093/eurheartj/ehac584. 36282295 10.1093/eurheartj/ehac584

[8] Ding J, Davis-Plourde KL, Sedaghat S, Tully PJ, Wang W, Phillips C, et al. Antihypertensive medications and risk for incident dementia and Alzheimer’s disease: a meta-analysis of individual participant data from prospective cohort studies. Lancet Neurol. 2020;19:61–70. 31706889 10.1016/S1474-4422(19)30393-X PMC7391421

[9] Peters R, Yasar S, Anderson CS, Andrews S, Antikainen R, Arima H, et al. Investigation of antihypertensive class, dementia, and cognitive decline: A meta-analysis. Neurology. 2020;94:e267–81. 31827004 10.1212/WNL.0000000000008732 PMC7108807

[10] Hampel H, Lista S. Dementia: the rising global tide of cognitive impairment. Nat Rev Neurol. 2016;12:131–2. 26782338 10.1038/nrneurol.2015.250

[11] Rea F, Corrao G, Mancia G. Risk of Dementia During Antihypertensive Drug Therapy in the Elderly. J Am Coll Cardiol. 2024;83:1194–203. 38538198 10.1016/j.jacc.2024.01.030

[12] Li H, Wang M, Qian F, Wu Z, Liu W, Wang A, et al. Association between untreated and treated blood pressure levels and cognitive decline in community-dwelling middle-aged and older adults in China: a longitudinal study. Alzheimers Res Ther. 2024;16:104. 38730505 10.1186/s13195-024-01467-y PMC11083800

[13] Wagg E, Blyth FM, Cumming RG, Khalatbari-Soltani S. Socioeconomic position and healthy ageing: A systematic review of cross-sectional and longitudinal studies. Ageing Res Rev. 2021;69:101365. 34004378 10.1016/j.arr.2021.101365

[14] Steptoe A, Zaninotto P. Lower socioeconomic status and the acceleration of aging: An outcome-wide analysis. Proc Natl Acad Sci. 2020;117:14911–7. 32541023 10.1073/pnas.1915741117 PMC7334539

[15] Li R, Li R, Xie J, Chen J, Liu S, Pan A, et al. Associations of socioeconomic status and healthy lifestyle with incident early-onset and late-onset dementia: a prospective cohort study. Lancet Heal Longev. 2023;4:e693-702. 10.1016/S2666-7568(23)00211-8 38042162

[16] Alsabbagh MHDW, Lemstra M, Eurich D, Lix LM, Wilson TW, Watson E, et al. Socioeconomic status and nonadherence to antihypertensive drugs: a systematic review and meta-analysis. Value Heal. 2014;17:288–96. 10.1016/j.jval.2013.11.011 24636389

[17] Zhao Y, Hu Y, Smith JP, Strauss J, Yang G. Cohort profile: the China Health and Retirement Longitudinal Study (CHARLS). Int J Epidemiol. 2014;43:61–8. 23243115 10.1093/ije/dys203 PMC3937970

[18] Whelton PK, Carey RM, Aronow WS, Casey DEJ, Collins KJ, Dennison Himmelfarb C, et al. 2017 ACC/AHA/AAPA/ABC/ACPM/AGS/APhA/ASH/ASPC/NMA/PCNA Guideline for the Prevention, Detection, Evaluation, and Management of High Blood Pressure in Adults: Executive Summary: A Report of the American College of Cardiology/American Heart Association Task F. Hypertension. 2018;71:1269–324. 29133354 10.1161/HYP.0000000000000066

[19] Li C, Ma Y, Yang C, Hua R, Xie W, Zhang L. Association of Cystatin C Kidney Function Measures With Long-term Deficit-Accumulation Frailty Trajectories and Physical Function Decline. JAMA Netw Open. 2022;5:e2234208. 36178684 10.1001/jamanetworkopen.2022.34208 PMC9526088

[20] Zhang Y-B, Chen C, Pan X-F, Guo J, Li Y, Franco OH, et al. Associations of healthy lifestyle and socioeconomic status with mortality and incident cardiovascular disease: two prospective cohort studies. BMJ. 2021;373:n604. 33853828 10.1136/bmj.n604 PMC8044922

[21] Ma Y, Li C, Hua R, Yang C, Xie W, Zhang L. Association Between Serum Cystatin C and Cognitive Decline Independently from Creatinine: Evidence from Two Nationally Representative Aging Cohorts. J Alzheimers Dis. 2023;93:459–69. 37038817 10.3233/JAD-221162

[22] Li C, Ma Y, Hua R, Zheng F, Xie W. Long-term physical activity participation trajectories were associated with subsequent cognitive decline, risk of dementia and all-cause mortality among adults aged ≥50 years: a population-based cohort study. Age Ageing. 2022;51:afac071. 35348603 10.1093/ageing/afac071

[23] Li C, Ma Y, Hua R, Yang Z, Zhong B, Wang H, et al. Dose-Response Relationship Between Long-Term Blood Pressure Variability and Cognitive Decline. Stroke. 2021;52:3249–57. 34167328 10.1161/STROKEAHA.120.033697

[24] Xie W, Zheng F, Yan L, Zhong B. Cognitive decline before and after incident coronary events. J Am Coll Cardiol. 2019;73:3041–50. 31221251 10.1016/j.jacc.2019.04.019

[25] Hua R, Ma Y, Li C, Zhong B, Xie W. Low levels of low-density lipoprotein cholesterol and cognitive decline. Sci Bull. 2021;66:1684–90. 10.1016/j.scib.2021.02.018 36654302

[26] Jia J, Zhao T, Liu Z, Liang Y, Li F, Li Y, et al. Association between healthy lifestyle and memory decline in older adults: 10 year, population based, prospective cohort study. BMJ. 2023;380:e072691. 36696990 10.1136/bmj-2022-072691 PMC9872850

[27] Ahmadi-Abhari S, Guzman-Castillo M, Bandosz P, Shipley MJ, Muniz-Terrera G, Singh-Manoux A, et al. Temporal trend in dementia incidence since 2002 and projections for prevalence in England and Wales to 2040: modelling study. BMJ. 2017;358:j2856. 28679494 10.1136/bmj.j2856 PMC5497174

[28] Geneletti S, Mason A, Best N. Adjusting for selection effects in epidemiologic studies: why sensitivity analysis is the only “solution.” Epidemiology. 2011;22:36–9. 21150353 10.1097/EDE.0b013e3182003276

[29] Imai K, Ratkovic M. Covariate balancing propensity score. J R Stat Soc Ser B (Statistical Methodol). 2014;76:243–63.

[30] Cho MH, Shin DW, Chang S-A, Lee JE, Jeong S-M, Kim SH, et al. Association between cognitive impairment and poor antihypertensive medication adherence in elderly hypertensive patients without dementia. Sci Rep. 2018;8:11688. 30076332 10.1038/s41598-018-29974-7 PMC6076290

[31] Chou C-C, Chien L-Y, Liaw J-J, Wang C-J, Liu P-Y. Association between cognitive function and self-reported antihypertensive medication adherence among middle-aged and older hypertensive women. J Clin Nurs. 2022;31:2839–49. 34723423 10.1111/jocn.16106

[32] McMaughan DJ, Oloruntoba O, Smith ML. Socioeconomic Status and Access to Healthcare: Interrelated Drivers for Healthy Aging. Front public Heal. 2020;8:231. 10.3389/fpubh.2020.00231 PMC7314918 32626678

[33] Shi L, Tao L, Chen N, Liang H. Relationship between socioeconomic status and cognitive ability among Chinese older adults: the moderating role of social support. Int J Equity Health. 2023;22:70. 37095501 10.1186/s12939-023-01887-6 PMC10124054

[34] Cadar D, Lassale C, Davies H, Llewellyn DJ, Batty GD, Steptoe A. Individual and Area-Based Socioeconomic Factors Associated With Dementia Incidence in England: Evidence From a 12-Year Follow-up in the English Longitudinal Study of Ageing. JAMA Psychiat. 2018;75:723–32. 10.1001/jamapsychiatry.2018.1012 PMC6145673 29799983

[35] Petersen JD, Wehberg S, Packness A, Svensson NH, Hyldig N, Raunsgaard S, et al. Association of Socioeconomic Status With Dementia Diagnosis Among Older Adults in Denmark. JAMA Netw open. 2021;4:e2110432. 34003271 10.1001/jamanetworkopen.2021.10432 PMC8132141

[36] Muir RT, Hill MD, Black SE, Smith EE. Minimal clinically important difference in Alzheimer’s disease: Rapid review. Alzheimers Dement. 2024;20:3352–63. 38561021 10.1002/alz.13770 PMC11095473

[37] Streeter AJ, Lin NX, Crathorne L, Haasova M, Hyde C, Melzer D, et al. Adjusting for unmeasured confounding in non-randomised longitudinal studies: a methodological review. J Clin Epidemiol. 2017;87:23–34. 28460857 10.1016/j.jclinepi.2017.04.022 PMC5589113

