# Supplementary material for: Anti-hypertensive medication adherence, socioeconomic status, and cognitive aging in the Chinese community-dwelling middle-aged and older adults ≥ 45 years: a population-based longitudinal study
Source: BMC Med. 2025 Feb 25;23:121. doi: 10.1186/s12916-025-03949-8 (PMC11863513; doi:10.1186/s12916-025-03949-8)
Supplement: Supplementary file 1 — Additional file 1: Supplemental Methods. Details of cognitive function assessment in the CHARLS. Table S1. Item-response probabilities for the latent class analysis for classifying socioeconomic status. Table S2. Comparison of socioeconomic status indicators by identified latent classes. Table S3. Baseline characteristics of participants by longitudinal adherence to anti-hypertensive medication. Table S4. Associations between anti-hypertensive medication use and the annual rate of change in cognitive function, further excluding individuals with reported memory-related disease or developed cognitive impairment during wave 1 and wave 2. Table S5. Associations between anti-hypertensive medication use and the annual rate of change in cognitive function, further accounting for the use of Chinese traditional medicine. Table S6. Associations between anti-hypertensive medication use and annual rate of change in cognitive function, accounting for other cognitive domains. Table S7. Associations between anti-hypertensive medication use and annual rate of change in cognitive function, further controlling for blood pressure follow-up measurements. Table S8. Associations between anti-hypertensive medication use and the annual rate of change in cognitive function, controlling for the overall socioeconomic status. Table S9. Associations between anti-hypertensive medication use and the annual rate of change in cognitive function, controlling for all indicators of socioeconomic status. Table S10. Associations between overall socioeconomic status and the annual rate of change in cognitive function, controlling for anti-hypertensive medication use. Table S11. Associations between baseline cognition and follow-up cognitive changes with the adherence to anti-hypertensive medication in participants with hypertension. Table S12. Associations between longitudinal anti-hypertensive medication adherence and the annual rate of change in cognitive function, incorporating data from wave 1 to wav [file 12916_2025_3949_MOESM1_ESM.docx]

**Supplementary Methods**

**1. Cognitive function assessment**

Cognitive assessment was conducted in face-to-face interviews and covered three domains: memory, executive function, and orientation in the CHARLS cohort.

- 1. *Memory function*

The interviewers read out a set of 10 words “at a slow steady rate approximately one word every 2 seconds” and then ask the individual to “recall aloud as many of the words as you can, in any order”. The respondents had 2 minutes to complete the immediate recall test. After questions and tests on other aspects, the participants were again asked to recall the words as much as they could. For each word correctly recalled (no matter in immediate or delayed recall), 1 point was assigned, with the total score ranging from 0 to 20.

- 1. *Executive function*

In the CHARLS, participants were asked to answer “What does 100 minus 7 equal? And 7 from that? And 7 from that? And 7 from that? And 7 from that?” This test was denoted as serial 7 test, to partially reflect the executive function of the participants. Correct subtractions are based on the previous answer given, even if the previous answer was incorrect. For each correct subtraction, 1 point was assigned, with the total score ranging from 0 to 5.

- 1. *Orientation*

The participants were asked to report “today's date (the day of month, month, year)” and “what day of the week it is today”. For each correct answer, 1 point was assigned, with the total score ranging from 0 to 4.

**Supplementary Tables and Supplementary Figures**

[**Table S1.** Item-response probabilities for the latent class analysis for classifying socioeconomic status.](#_Toc184657592)

[**Table S2.** Comparison of socioeconomic status indicators by identified latent classes.](#_Toc184657593)

[**Table S3.** Baseline characteristics of participants by longitudinal adherence to anti-hypertensive medication.](#_Toc184657594)

[**Table S4.** Associations between anti-hypertensive medication use and the annual rate of change in cognitive function, further excluding individuals with reported memory-related disease or developed cognitive impairment during wave 1 and wave 2.](#_Toc184657595)

[**Table S5.** Associations between anti-hypertensive medication use and the annual rate of change in cognitive function, further accounting for the use of Chinese traditional medicine.](#_Toc184657596)

[**Table S6.** Associations between anti-hypertensive medication use and annual rate of change in cognitive function, accounting for other cognitive domains.](#_Toc184657597)

[**Table S7.** Associations between anti-hypertensive medication use and annual rate of change in cognitive function, further controlling for blood pressure follow-up measurements.](#_Toc184657598)

[**Table S8.** Associations between anti-hypertensive medication use and the annual rate of change in cognitive function, controlling for the overall socioeconomic status.](#_Toc184657599)

[**Table S9.** Associations between anti-hypertensive medication use and the annual rate of change in cognitive function, controlling for all indicators of socioeconomic status.](#_Toc184657600)

[**Table S10.** Associations between overall socioeconomic status and the annual rate of change in cognitive function, controlling for anti-hypertensive medication use.](#_Toc184657601)

[**Table S11.** Associations between baseline cognition and follow-up cognitive changes with the adherence to anti-hypertensive medication in participants with hypertension.](#_Toc184657602)

[**Table S12.** Associations between longitudinal anti-hypertensive medication adherence and the annual rate of change in cognitive function, incorporating data from wave 1 to wave 4.](#_Toc184657603)

[**Table S13.** Associations between anti-hypertensive medication use and the annual rate of change in cognitive function, based on inverse probability-weighted samples.](#_Toc184657604)

[**Table S14.** Baseline characteristics comparison between participants included and excluded from analysis in CHARLS.](#_Toc184657605)

**Figure S1.** Participants selection diagram.

**Figure S2.** Joint associations of baseline anti-hypertensive medication use and socioeconomic status with declines in global cognition.

**Figure S3.** Joint associations of baseline anti-hypertensive medication use and socioeconomic status with declines in memory function.

**Figure S4.** Joint associations of longitudinal anti-hypertensive medication adherence and socioeconomic status with declines in global cognition.

**Figure S5.** Joint associations of longitudinal anti-hypertensive medication adherence and socioeconomic status with declines in memory function.

**Figure S6.** Love plot assessing the difference in baseline characteristics between participants included in and excluded from the primary analysis, before and after inverse probability weighting.

**Figure S7.** Joint associations of baseline anti-hypertensive medication use and baseline age with cognitive aging.

**Figure S8.** Joint associations of longitudinal anti-hypertensive medication adherence and baseline age with cognitive aging.

**Table S1.** Item-response probabilities for the latent class analysis for classifying socioeconomic status.

| **Items for LCA** | **Socioeconomic status classes in CHARLS (N=9229) ^a^** | |
| --- | --- | --- |
|  | **Latent class 1 (N=8375)** | **Latent class 2 (N=854)** |
| **Education** | | |
| Less than high school | 0.93 | 0.62 |
| High school or equivalent | 0.07 | 0.31 |
| College and above | 0.00 | 0.07 |
| **Family annual income** | | |
| Below the median | 0.62 | 0.00 |
| Above the median | 0.38 | 1.00 |
| **Employment status** | | |
| Unemployed | 0.03 | 0.02 |
| Employed | 0.97 | 0.98 |
| **Medical insurance coverage** | | |
| Uninsured | 0.07 | 0.04 |
| Insured | 0.93 | 0.96 |

^a^ The latent classes were derived using the selected CHARLS sample (N=9229). LCA, latent class analysis; CHARLS, China Health and Retirement Longitudinal Study.

**Table S2.** Comparison of socioeconomic status indicators by identified latent classes.

| **Socioeconomic status indicators, n (%)** | **Socioeconomic status classes in CHARLS (N=9229) ^a^** | | ***P* value ^b^** |
| --- | --- | --- | --- |
|  | **Lower class (N=8375)** | **Upper class (N=854)** |  |
| **Education** | | | <0.001 |
| Less than high school | 8066 (96.3) | 0 (0.0) |  |
| High school or equivalent | 300 (3.6) | 720 (84.3) |  |
| College and above | 9 (0.1) | 134 (15.7) |  |
| **Family annual income** | | | <0.001 |
| Below the median | 4614 (55.1) | 0 (0.0) |  |
| Above the median | 3761 (44.9) | 854 (100.0) |  |
| **Employment status** | | | 0.933 |
| Unemployed | 245 (2.9) | 24 (2.8) |  |
| Employed | 8130 (97.1) | 830 (97.2) |  |
| **Medical insurance coverage** | | | 0.042 |
| Uninsured | 536 (6.4) | 39 (4.6) |  |
| Insured | 7839 (93.6) | 815 (95.4) |  |

^a^ The latent classes were derived using the selected CHARLS sample (N=9229). CHARLS, China Health and Retirement Longitudinal Study.

^b^ The difference in socioeconomic status indicators between identified classes was examined using chi-squared test.

**Table S3.** Baseline characteristics of participants by longitudinal adherence to anti-hypertensive medication.

| **Characteristics** | **Participants, No. (%)** | | | | | ***P* value ^a^** |
| --- | --- | --- | --- | --- | --- | --- |
|  | **All participants**  **N=9229** | **With hypertension** | | | **Normotension**  **N=5631** |  |
|  |  | **Low adherence**  **N=1588** | **Moderate adherence**  **N=858** | **High adherence**  **N=1152** |  |  |
| Age, mean (SD), y | 57.1 (8.9) | 59.1 (9.4) | 59.3 (8.9) | 60.4 (8.4) | 55.4 (8.4) | <0.001 |
| Sex |  |  |  |  |  | <0.001 |
| Men | 4687 (50.8) | 875 (55.1) | 420 (49.0) | 530 (46.0) | 2862 (50.8) |  |
| Women | 4542 (49.2) | 713 (44.9) | 438 (51.0) | 622 (54.0) | 2769 (49.2) |  |
| Overall socioeconomic status ^b^ | | | | | | 0.015 |
| Lower | 8375 (90.7) | 1473 (92.8) | 782 (91.1) | 1045 (90.7) | 5075 (90.1) |  |
| Upper | 854 (9.3) | 115 (7.2) | 76 (8.9) | 107 (9.3) | 556 (9.9) |  |
| Education |  |  |  |  |  | 0.065 |
| Less than high school | 8066 (87.4) | 1423 (89.6) | 755 (88.0) | 1012 (87.8) | 4876 (86.6) |  |
| High school or equivalent | 1020 (11.1) | 141 (8.9) | 90 (10.5) | 122 (10.6) | 667 (11.8) |  |
| College and higher | 143 (1.5) | 24 (1.5) | 13 (1.5) | 18 (1.6) | 88 (1.6) |  |
| Annual family income ^c^ |  |  |  |  |  | <0.001 |
| Below the median | 4614 (50.0) | 871 (54.8) | 436 (50.8) | 582 (50.5) | 2725 (48.4) |  |
| Above the median | 4615 (50.0) | 717 (45.2) | 422 (49.2) | 570 (49.5) | 2906 (51.6) |  |
| Medical insurance coverage |  |  |  |  |  | 0.164 |
| Uninsured | 575 (6.2) | 114 (7.2) | 58 (6.8) | 60 (5.2) | 343 (6.1) |  |
| Insured | 8654 (93.8) | 1474 (92.8) | 800 (93.2) | 1092 (94.8) | 5288 (93.9) |  |
| Employment status |  |  |  |  |  | 0.003 |
| Unemployed | 269 (2.9) | 38 (2.4) | 34 (4.0) | 49 (4.3) | 148 (2.6) |  |
| Employed | 8960 (97.1) | 1550 (97.6) | 824 (96.0) | 1103 (95.7) | 5483 (97.4) |  |
| Living alone | 881 (9.5) | 212 (13.4) | 97 (11.3) | 139 (12.1) | 433 (7.7) | <0.001 |
| Physical exercise | 2721 (29.5) | 455 (28.7) | 209 (24.4) | 290 (25.2) | 1767 (31.4) | <0.001 |
| Alcohol consumption | 1623 (17.6) | 323 (20.3) | 165 (19.2) | 143 (12.4) | 992 (17.6) | <0.001 |
| Current smoking | 3004 (32.5) | 590 (37.2) | 259 (30.2) | 276 (24.0) | 1879 (33.4) | <0.001 |
| Systolic blood pressure, mean (SD), mmHg | 128.4 (20.8) | 147.0 (17.2) | 146.4 (22.2) | 143.6 (21.6) | 117.3 (11.6) | <0.001 |
| Diastolic blood pressure, mean (SD), mmHg | 75.5 (12.1) | 84.7 (11.4) | 83.6 (12.9) | 81.9 (12.5) | 70.4 (8.9) | <0.001 |
| Physical disability | 1046 (11.3) | 176 (11.1) | 126 (14.7) | 185 (16.1) | 559 (9.9) | <0.001 |
| Hypertension | 3598 (39.0) | 1588 (100.0) | 858 (100.0) | 1152 (100.0) | 0 (0.0) | <0.001 |
| Diabetes | 564 (6.1) | 81 (5.1) | 93 (10.8) | 184 (16.0) | 206 (3.7) | <0.001 |
| Cancer | 78 (0.8) | 12 (0.8) | 10 (1.2) | 12 (1.0) | 44 (0.8) | 0.571 |
| Chronic lung disease | 858 (9.3) | 172 (10.8) | 81 (9.4) | 117 (10.2) | 488 (8.7) | 0.044 |
| Heart disease | 1071 (11.6) | 144 (9.1) | 163 (19.0) | 320 (27.8) | 444 (7.9) | <0.001 |
| Stroke | 189 (2.0) | 28 (1.8) | 32 (3.7) | 65 (5.6) | 64 (1.1) | <0.001 |
| Kidney disease | 496 (5.4) | 85 (5.4) | 66 (7.7) | 62 (5.4) | 283 (5.0) | 0.015 |
| Memory function score, mean (SD) | 7.6 (3.3) | 7.4 (3.3) | 7.4 (3.2) | 7.3 (3.0) | 7.7 (3.3) | <0.001 |
| Executive function score, median (IQR) | 4.0 (3.0-5.0) | 4.0 (3.0-5.0) | 4.0 (3.0-5.0) | 4.0 (3.0-5.0) | 4.0 (3.0-5.0) | 0.015 |
| Orientation score, median (IQR) | 3.0 (3.0-4.0) | 3.0 (3.0-4.0) | 3.0 (3.0-4.0) | 4.0 (3.0-4.0) | 3.0 (3.0-4.0) | 0.001 |

Abbreviations: SD, standard deviation; IQR, interquartile range.

^a^ P value reported for differences between groups using analysis of variance, Kruskal-Wallis test, or chi-squared test.

^b^ Latent class analysis was used for classifying the overall socioeconomic status, derived using indicators of education, income, employment status, and medical insurance.

^c^ Median of annual family income was $2877.7 (to convert to Chinese yuan, multiplied by 6.3).

**Table S4.** Associations between anti-hypertensive medication use and the annual rate of change in cognitive function, further excluding individuals with reported memory-related disease or developed cognitive impairment during wave 1 and wave 2.

| **Anti-hypertensive medication** | **No. of participants** | **Global cognition (SD/y)** | |  | **Memory function (SD/y)** | |
| --- | --- | --- | --- | --- | --- | --- |
|  |  | **β (95% CI) ^a^** | ***P* value** |  | **β (95% CI) ^a^** | ***P* value** |
| Baseline anti-hypertensive medication use | | | | | | |
| Hypertension not using medication | 1738 | 0 [Reference] | NA |  | 0 [Reference] | NA |
| Hypertension using medication | 1333 | 0.014 (0.003, 0.025) | 0.016 |  | 0.022 (0.006, 0.037) | 0.007 |
| Normotension | 4947 | 0.020 (0.011, 0.028) | <0.001 |  | 0.029 (0.017, 0.041) | <0.001 |
| Compared to normotension |  |  |  |  |  |  |
| Normotension | 4947 | 0 [Reference] | NA |  | 0 [Reference] | NA |
| Hypertension not using medication | 1738 | -0.020 (-0.028, -0.011) | <0.001 |  | -0.029 (-0.041, -0.017) | <0.001 |
| Hypertension using medication | 1333 | -0.006 (-0.015, 0.004) | 0.239 |  | -0.007 (-0.020, 0.006) | 0.281 |
| Longitudinal adherence to anti-hypertensive medication | | | | | | |
| Hypertension, low adherence | 1337 | 0 [Reference] | NA |  | 0 [Reference] | NA |
| Hypertension, moderate adherence | 734 | 0.007 (-0.011, 0.024) | 0.460 |  | 0.006 (-0.014, 0.026) | 0.557 |
| Hypertension, high adherence | 1000 | 0.015 (-0.001, 0.031) | 0.064 |  | 0.024 (0.006, 0.042) | 0.008 |
| Normotension | 4947 | 0.020 (0.009, 0.032) | <0.001 |  | 0.029 (0.016, 0.042) | <0.001 |
| Compared to normotension |  |  |  |  |  |  |
| Normotension | 4947 | 0 [Reference] | NA |  | 0 [Reference] | NA |
| Hypertension, low adherence | 1337 | -0.020 (-0.032, -0.009) | <0.001 |  | -0.029 (-0.042, -0.016) | <0.001 |
| Hypertension, moderate adherence | 734 | -0.014 (-0.029, 0.001) | 0.075 |  | -0.023 (-0.040, -0.006) | 0.008 |
| Hypertension, high adherence | 1000 | -0.005 (-0.018, 0.008) | 0.445 |  | -0.005 (-0.019, 0.010) | 0.552 |

NA, not applicable.

^a^ β coefficient was estimated using linear mixed models, with a positive value representing decelerated cognitive decline in comparison.

Adjusted covariates included age, sex, education, cohabitation status, physical activity, alcohol consumption, current smoking, physical disability, hypertension, diabetes, cancer, chronic lung disease, heart disease, stroke, kidney disease, and baseline measurements of blood pressure (systolic and diastolic blood pressure).

**Table S5.** Associations between anti-hypertensive medication use and the annual rate of change in cognitive function, further accounting for the use of Chinese traditional medicine.

| **Anti-hypertensive medication** | **No. of participants** | **Global cognition (SD/y)** | |  | **Memory function (SD/y)** | |
| --- | --- | --- | --- | --- | --- | --- |
|  |  | **β (95% CI) ^a^** | ***P* value** |  | **β (95% CI) ^a^** | ***P* value** |
| Baseline anti-hypertensive medication use | | | | | | |
| Hypertension not using medication | 1968 | 0 [Reference] | NA |  | 0 [Reference] | NA |
| Hypertension using medication | 1630 | 0.015 (0.004, 0.026) | 0.008 |  | 0.020 (0.007, 0.033) | 0.002 |
| Normotension | 5631 | 0.018 (0.010, 0.027) | <0.001 |  | 0.028 (0.018, 0.038) | <0.001 |
| Compared to normotension |  |  |  |  |  |  |
| Normotension | 5631 | 0 [Reference] | NA |  | 0 [Reference] | NA |
| Hypertension not using medication | 1968 | -0.018 (-0.027, -0.010) | <0.001 |  | -0.028 (-0.038, -0.018) | <0.001 |
| Hypertension using medication | 1630 | -0.004 (-0.013, 0.005) | 0.432 |  | -0.008 (-0.019, 0.003) | 0.141 |
| Longitudinal adherence to anti-hypertensive medication | | | | | | |
| Hypertension, low adherence | 1525 | 0 [Reference] | NA |  | 0 [Reference] | NA |
| Hypertension, moderate adherence | 789 | 0.012 (-0.002, 0.027) | 0.089 |  | 0.010 (-0.007, 0.027) | 0.238 |
| Hypertension, high adherence | 1284 | 0.013 (0.001, 0.026) | 0.036 |  | 0.020 (0.006, 0.035) | 0.007 |
| Normotension | 5631 | 0.019 (0.010, 0.029) | <0.001 |  | 0.028 (0.017, 0.040) | <0.001 |
| Compared to normotension |  |  |  |  |  |  |
| Normotension | 5631 | 0 [Reference] | NA |  | 0 [Reference] | NA |
| Hypertension, low adherence | 1525 | -0.019 (-0.029, -0.010) | <0.001 |  | -0.028 (-0.040, -0.017) | <0.001 |
| Hypertension, moderate adherence | 789 | -0.007 (-0.019, 0.006) | 0.282 |  | -0.018 (-0.033, -0.004) | 0.014 |
| Hypertension, high adherence | 1284 | -0.006 (-0.016, 0.004) | 0.248 |  | -0.008 (-0.020, 0.004) | 0.175 |

NA, not applicable.

^a^ β coefficient was estimated using linear mixed models, with a positive value representing decelerated cognitive decline in comparison.

Adjusted covariates included age, sex, education, cohabitation status, physical activity, alcohol consumption, current smoking, physical disability, hypertension, diabetes, cancer, chronic lung disease, heart disease, stroke, kidney disease, and baseline measurements of blood pressure (systolic and diastolic blood pressure).

**Table S6.** Associations between anti-hypertensive medication use and annual rate of change in cognitive function, accounting for other cognitive domains.

| **Anti-hypertensive medication** | **No. of participants** | **Executive function (SD/y)** | |  | **Orientation (SD/y)** | |
| --- | --- | --- | --- | --- | --- | --- |
|  |  | **β (95% CI) ^a^** | ***P* value** |  | **β (95% CI) ^a^** | ***P* value** |
| Baseline anti-hypertensive medication use | | | | | | |
| Hypertension not using medication | 1968 | 0 [Reference] | NA |  | 0 [Reference] | NA |
| Hypertension using medication | 1630 | 0.010 (-0.001, 0.021) | 0.083 |  | -0.004 (-0.016, 0.008) | 0.531 |
| Normotension | 5631 | 0.006 (-0.003, 0.014) | 0.183 |  | 0.007 (-0.002, 0.016) | 0.140 |
| Compared to normotension |  |  |  |  |  |  |
| Normotension | 5631 | 0 [Reference] | NA |  | 0 [Reference] | NA |
| Hypertension not using medication | 1968 | -0.006 (-0.014, 0.003) | 0.183 |  | -0.007 (-0.016, 0.002) | 0.140 |
| Hypertension using medication | 1630 | 0.004 (-0.005, 0.014) | 0.393 |  | -0.010 (-0.020, -0.000) | 0.040 |
| Longitudinal adherence to anti-hypertensive medication | | | | | | |
| Hypertension, low adherence | 1525 | 0 [Reference] | NA |  | 0 [Reference] | NA |
| Hypertension, moderate adherence | 789 | 0.009 (-0.005, 0.023) | 0.195 |  | 0.009 (-0.009, 0.027) | 0.329 |
| Hypertension, high adherence | 1284 | 0.005 (-0.008, 0.018) | 0.440 |  | 0.003 (-0.014, 0.019) | 0.726 |
| Normotension | 5631 | 0.005 (-0.004, 0.015) | 0.265 |  | 0.012 (-0.000, 0.024) | 0.052 |
| Compared to normotension |  |  |  |  |  |  |
| Normotension | 5631 | 0 [Reference] | NA |  | 0 [Reference] | NA |
| Hypertension, low adherence | 1525 | -0.005 (-0.015, 0.004) | 0.265 |  | -0.012 (-0.024, 0.000) | 0.052 |
| Hypertension, moderate adherence | 789 | 0.004 (-0.008, 0.016) | 0.521 |  | -0.003 (-0.018, 0.013) | 0.714 |
| Hypertension, high adherence | 1284 | -0.000 (-0.011, 0.010) | 0.960 |  | -0.009 (-0.023, 0.005) | 0.202 |

NA, not applicable.

^a^ β coefficient was estimated using linear mixed models, with a positive value representing decelerated cognitive decline in comparison.

Adjusted covariates included age, sex, education, cohabitation status, physical activity, alcohol consumption, current smoking, physical disability, hypertension, diabetes, cancer, chronic lung disease, heart disease, stroke, kidney disease, and baseline measurements of blood pressure (systolic and diastolic blood pressure).

**Table S7.** Associations between anti-hypertensive medication use and annual rate of change in cognitive function, further controlling for blood pressure follow-up measurements.

| **Anti-hypertensive medication** | **No. of participants** | **Global cognition (SD/y)** | |  | **Memory function (SD/y)** | |
| --- | --- | --- | --- | --- | --- | --- |
|  |  | **β (95% CI) ^a^** | ***P* value** |  | **β (95% CI) ^a^** | ***P* value** |
| Baseline anti-hypertensive medication use | | | | | | |
| Hypertension not using medication | 1520 | 0 [Reference] | NA |  | 0 [Reference] | NA |
| Hypertension using medication | 1101 | 0.016 (0.004, 0.029) | 0.012 |  | 0.022 (0.007, 0.037) | 0.005 |
| Normotension | 4145 | 0.022 (0.013, 0.032) | <0.001 |  | 0.028 (0.016, 0.039) | <0.001 |
| Compared to normotension |  |  |  |  |  |  |
| Normotension | 4145 | 0 [Reference] | NA |  | 0 [Reference] | NA |
| Hypertension not using medication | 1520 | -0.022 (-0.032, -0.013) | <0.001 |  | -0.028 (-0.039, -0.016) | <0.001 |
| Hypertension using medication | 1101 | -0.006 (-0.017, 0.005) | 0.285 |  | -0.006 (-0.019, 0.007) | 0.362 |
| Longitudinal adherence to anti-hypertensive medication | | | | | | |
| Hypertension, low adherence | 1148 | 0 [Reference] | NA |  | 0 [Reference] | NA |
| Hypertension, moderate adherence | 596 | 0.019 (0.002, 0.035) | 0.027 |  | 0.005 (-0.015, 0.024) | 0.643 |
| Hypertension, high adherence | 877 | 0.020 (0.006, 0.035) | 0.006 |  | 0.024 (0.006, 0.041) | 0.008 |
| Normotension | 4145 | 0.027 (0.016, 0.037) | <0.001 |  | 0.028 (0.015, 0.040) | <0.001 |
| Compared to normotension |  |  |  |  |  |  |
| Normotension | 4145 | 0 [Reference] | NA |  | 0 [Reference] | NA |
| Hypertension, low adherence | 1148 | -0.027 (-0.037, -0.016) | <0.001 |  | -0.028 (-0.040, -0.015) | <0.001 |
| Hypertension, moderate adherence | 596 | -0.008 (-0.022, 0.006) | 0.271 |  | -0.023 (-0.040, -0.006) | 0.007 |
| Hypertension, high adherence | 877 | -0.006 (-0.018, 0.006) | 0.318 |  | -0.004 (-0.018, 0.010) | 0.581 |

NA, not applicable.

^a^ β coefficient was estimated using linear mixed models, with a positive value representing decelerated cognitive decline in comparison.

Adjusted covariates included age, sex, education, cohabitation status, physical activity, alcohol consumption, current smoking, physical disability, hypertension, diabetes, cancer, chronic lung disease, heart disease, stroke, kidney disease, and baseline measurements of blood pressure (systolic and diastolic blood pressure). Follow-up measurements of systolic and diastolic blood pressure were simultaneously adjusted.

**Table S8.** Associations between anti-hypertensive medication use and the annual rate of change in cognitive function, controlling for the overall socioeconomic status.

| **Anti-hypertensive medication** | **No. of participants** | **Global cognition (SD/y)** | |  | **Memory function (SD/y)** | |
| --- | --- | --- | --- | --- | --- | --- |
|  |  | **β (95% CI) ^a^** | ***P* value** |  | **β (95% CI) ^a^** | ***P* value** |
| Baseline anti-hypertensive medication use | | | | | | |
| Hypertension not using medication | 2061 | 0 [Reference] | NA |  | 0 [Reference] | NA |
| Hypertension using medication | 1537 | 0.014 (0.003, 0.025) | 0.011 |  | 0.021 (0.008, 0.034) | 0.001 |
| Normotension | 5631 | 0.018 (0.010, 0.026) | <0.001 |  | 0.028 (0.018, 0.038) | <0.001 |
| Compared to normotension |  |  |  |  |  |  |
| Normotension | 5631 | 0 [Reference] | NA |  | 0 [Reference] | NA |
| Hypertension not using medication | 2061 | -0.018 (-0.026, -0.010) | <0.001 |  | -0.028 (-0.038, -0.018) | <0.001 |
| Hypertension using medication | 1537 | -0.004 (-0.013, 0.006) | 0.436 |  | -0.007 (-0.018, 0.004) | 0.219 |
| Longitudinal adherence to anti-hypertensive medication | | | | | | |
| Hypertension, low adherence | 1588 | 0 [Reference] | NA |  | 0 [Reference] | NA |
| Hypertension, moderate adherence | 858 | 0.012 (-0.002, 0.026) | 0.088 |  | 0.006 (-0.010, 0.023) | 0.449 |
| Hypertension, high adherence | 1152 | 0.014 (0.002, 0.027) | 0.025 |  | 0.023 (0.008, 0.038) | 0.002 |
| Normotension | 5631 | 0.019 (0.010, 0.029) | <0.001 |  | 0.028 (0.017, 0.039) | <0.001 |
| Compared to normotension |  |  |  |  |  |  |
| Normotension | 5631 | 0 [Reference] | NA |  | 0 [Reference] | NA |
| Hypertension, low adherence | 1588 | -0.019 (-0.029, -0.010) | <0.001 |  | -0.028 (-0.039, -0.017) | <0.001 |
| Hypertension, moderate adherence | 858 | -0.007 (-0.019, 0.005) | 0.231 |  | -0.022 (-0.036, -0.008) | 0.002 |
| Hypertension, high adherence | 1152 | -0.005 (-0.015, 0.006) | 0.365 |  | -0.005 (-0.017, 0.008) | 0.438 |

NA, not applicable.

^a^ β coefficient was estimated using linear mixed models, with a positive value representing decelerated cognitive decline in comparison.

Adjusted covariates included age, sex, cohabitation status, physical activity, alcohol consumption, current smoking, physical disability, hypertension, diabetes, cancer, chronic lung disease, heart disease, stroke, kidney disease, and baseline measurements of blood pressure (systolic and diastolic blood pressure). The latent class of overall socioeconomic status was also adjusted.

**Table S9.** Associations between anti-hypertensive medication use and the annual rate of change in cognitive function, controlling for all indicators of socioeconomic status.

| **Anti-hypertensive medication** | **No. of participants** | **Global cognition (SD/y)** | |  | **Memory function (SD/y)** | |
| --- | --- | --- | --- | --- | --- | --- |
|  |  | **β (95% CI) ^a^** | ***P* value** |  | **β (95% CI) ^a^** | ***P* value** |
| Baseline anti-hypertensive medication use | | | | | | |
| Hypertension not using medication | 2061 | 0 [Reference] | NA |  | 0 [Reference] | NA |
| Hypertension using medication | 1537 | 0.014 (0.003, 0.025) | 0.011 |  | 0.021 (0.008, 0.034) | 0.001 |
| Normotension | 5631 | 0.018 (0.009, 0.026) | <0.001 |  | 0.028 (0.018, 0.038) | <0.001 |
| Compared to normotension |  |  |  |  |  |  |
| Normotension | 5631 | 0 [Reference] | NA |  | 0 [Reference] | NA |
| Hypertension not using medication | 2061 | -0.018 (-0.026, -0.009) | <0.001 |  | -0.028 (-0.038, -0.018) | <0.001 |
| Hypertension using medication | 1537 | -0.004 (-0.013, 0.006) | 0.442 |  | -0.007 (-0.018, 0.004) | 0.228 |
| Longitudinal adherence to anti-hypertensive medication | | | | | | |
| Hypertension, low adherence | 1588 | 0 [Reference] | NA |  | 0 [Reference] | NA |
| Hypertension, moderate adherence | 858 | 0.012 (-0.002, 0.026) | 0.088 |  | 0.006 (-0.010, 0.023) | 0.449 |
| Hypertension, high adherence | 1152 | 0.014 (0.002, 0.027) | 0.025 |  | 0.023 (0.008, 0.038) | 0.003 |
| Normotension | 5631 | 0.019 (0.010, 0.028) | <0.001 |  | 0.028 (0.017, 0.039) | <0.001 |
| Compared to normotension |  |  |  |  |  |  |
| Normotension | 5631 | 0 [Reference] | NA |  | 0 [Reference] | NA |
| Hypertension, low adherence | 1588 | -0.019 (-0.028, -0.010) | <0.001 |  | -0.028 (-0.039, -0.017) | <0.001 |
| Hypertension, moderate adherence | 858 | -0.007 (-0.019, 0.005) | 0.240 |  | -0.022 (-0.036, -0.008) | 0.003 |
| Hypertension, high adherence | 1152 | -0.005 (-0.015, 0.006) | 0.371 |  | -0.005 (-0.017, 0.008) | 0.452 |

NA, not applicable.

^a^ β coefficient was estimated using linear mixed models, with a positive value representing decelerated cognitive decline in comparison.

Adjusted covariates included age, sex, education, cohabitation status, physical activity, alcohol consumption, current smoking, physical disability, hypertension, diabetes, cancer, chronic lung disease, heart disease, stroke, kidney disease, and baseline measurements of blood pressure (systolic and diastolic blood pressure). Family income, employment status, and medical insurance coverage were also adjusted.

**Table S10.** Associations between overall socioeconomic status and the annual rate of change in cognitive function, controlling for anti-hypertensive medication use.

| **Overall socioeconomic status** | **No. of participants** | **Global cognition (SD/y)** | |  | **Memory function (SD/y)** | |
| --- | --- | --- | --- | --- | --- | --- |
|  |  | **β (95% CI) ^a^** | ***P* value** |  | **β (95% CI) ^a^** | ***P* value** |
| Unadjusted for anti-hypertensive medication use | | | | | | |
| Lower | 8375 | 0 [Reference] | NA |  | 0 [Reference] | NA |
| Upper | 854 | 0.019 (0.008, 0.030) | <0.001 |  | 0.041 (0.028, 0.054) | <0.001 |
| Adjusted for baseline anti-hypertensive medication use | | | | | | |
| Lower | 8375 | 0 [Reference] | NA |  | 0 [Reference] | NA |
| Upper | 854 | 0.019 (0.008, 0.030) | <0.001 |  | 0.041 (0.028, 0.054) | <0.001 |
| Adjusted for longitudinal adherence to anti-hypertensive medication | | | | | | |
| Lower | 8375 | 0 [Reference] | NA |  | 0 [Reference] | NA |
| Upper | 854 | 0.019 (0.008, 0.030) | <0.001 |  | 0.041 (0.028, 0.054) | <0.001 |

NA, not applicable.

^a^ β coefficient was estimated using linear mixed models, with a positive value representing decelerated cognitive decline in comparison.

Adjusted covariates included age, sex, cohabitation status, physical activity, alcohol consumption, current smoking, physical disability, hypertension, diabetes, cancer, chronic lung disease, heart disease, stroke, kidney disease, and baseline measurements of blood pressure (systolic and diastolic blood pressure).

**Table S11.** Associations between baseline cognition and follow-up cognitive changes with the adherence to anti-hypertensive medication in participants with hypertension.

| **Cognitive scores** | **High longitudinal adherence to anti-hypertensive medication (n=1152)** | |
| --- | --- | --- |
|  | **Odds ratio (95% CI) ^a^** | ***P* value** |
| **Baseline global cognition score, wave 1** |  |  |
| Quartile 1 | 1 [Reference] | NA |
| Quartile 2 | 1.27 (1.02, 1.56) | 0.029 |
| Quartile 3 | 1.41 (1.14, 1.74) | 0.002 |
| Quartile 4 | 1.25 (1.00, 1.56) | 0.051 |
| Per SD increment | 1.09 (1.01, 1.18) | 0.026 |
| **Change in global cognition score, wave 1 and wave 2 ^b^** |  |  |
| Quartile 1 | 1 [Reference] | NA |
| Quartile 2 | 1.28 (1.02, 1.60) | 0.033 |
| Quartile 3 | 1.21 (0.97, 1.52) | 0.094 |
| Quartile 4 | 1.28 (1.00, 1.64) | 0.053 |
| Per SD increment | 1.13 (1.03, 1.25) | 0.011 |

NA, not applicable.

^a^ Odds ratio was estimated using binary logistic regression model, restricted to participants with hypertension (n=3598). Adjusted covariates included age, sex, cohabitation status, overall socioeconomic status, physical activity, alcohol consumption, current smoking, physical disability, hypertension, diabetes, cancer, chronic lung disease, heart disease, stroke, kidney disease, and baseline measurements of blood pressure (systolic and diastolic blood pressure).

^b^ Change in global cognition score was calculated as wave 2 score – wave 1 score. The baseline global cognition score was also adjusted in the model.

**Table S12.** Associations between longitudinal anti-hypertensive medication adherence and the annual rate of change in cognitive function, incorporating data from wave 1 to wave 4.

| **Anti-hypertensive medication** | **No. of participants** | **Global cognition (SD/y)** | |  | **Memory function (SD/y)** | |
| --- | --- | --- | --- | --- | --- | --- |
|  |  | **β (95% CI) ^a^** | ***P* value** |  | **β (95% CI) ^a^** | ***P* value** |
| Number of waves reporting anti-hypertensive medication use | | | | | | |
| Hypertension, 0 wave | 987 | 0 [Reference] | NA |  | 0 [Reference] | NA |
| Hypertension, 1 wave | 588 | 0.004 (-0.013, 0.021) | 0.660 |  | -0.006 (-0.027, 0.014) | 0.539 |
| Hypertension, 2 waves | 593 | 0.000 (-0.017, 0.018) | 0.996 |  | -0.004 (-0.025, 0.017) | 0.715 |
| Hypertension, 3 waves | 671 | 0.008 (-0.008, 0.024) | 0.335 |  | 0.009 (-0.011, 0.028) | 0.368 |
| Hypertension, 4 waves | 759 | 0.014 (-0.001, 0.030) | 0.069 |  | 0.020 (0.002, 0.038) | 0.032 |
| Normotension | 5631 | 0.017 (0.006, 0.029) | 0.003 |  | 0.024 (0.011, 0.037) | <0.001 |
| Compared to normotension |  |  |  |  |  |  |
| Normotension | 5631 | 0 [Reference] | NA |  | 0 [Reference] | NA |
| Hypertension, 0 wave | 987 | -0.017 (-0.029, -0.006) | 0.003 |  | -0.024 (-0.037, -0.011) | <0.001 |
| Hypertension, 1 wave | 588 | -0.014 (-0.028, 0.001) | 0.067 |  | -0.030 (-0.047, -0.013) | <0.001 |
| Hypertension, 2 waves | 593 | -0.017 (-0.032, -0.003) | 0.019 |  | -0.028 (-0.045, -0.011) | 0.002 |
| Hypertension, 3 waves | 671 | -0.009 (-0.023, 0.004) | 0.167 |  | -0.015 (-0.031, 0.001) | 0.060 |
| Hypertension, 4 waves | 759 | -0.003 (-0.015, 0.009) | 0.596 |  | -0.004 (-0.018, 0.010) | 0.572 |

NA, not applicable.

^a^ β coefficient was estimated using linear mixed models, with a positive value representing decelerated cognitive decline in comparison.

Adjusted covariates included age, sex, education, cohabitation status, physical activity, alcohol consumption, current smoking, physical disability, hypertension, diabetes, cancer, chronic lung disease, heart disease, stroke, kidney disease, and baseline measurements of blood pressure (systolic and diastolic blood pressure).

**Table S13.** Associations between anti-hypertensive medication use and the annual rate of change in cognitive function, based on inverse probability-weighted samples.

| **Anti-hypertensive medication** | **No. of participants** | **Global cognition (SD/y)** | |  | **Memory function (SD/y)** | |
| --- | --- | --- | --- | --- | --- | --- |
|  |  | **β (95% CI) ^a^** | ***P* value** |  | **β (95% CI) ^a^** | ***P* value** |
| Baseline anti-hypertensive medication use | | | | | | |
| Hypertension not using medication | 2061 | 0 [Reference] | NA |  | 0 [Reference] | NA |
| Hypertension using medication | 1537 | 0.013 (0.002, 0.025) | 0.020 |  | 0.020 (0.005, 0.035) | 0.009 |
| Normotension | 5631 | 0.019 (0.011, 0.028) | <0.001 |  | 0.030 (0.019, 0.042) | <0.001 |
| Compared to normotension |  |  |  |  |  |  |
| Normotension | 5631 | 0 [Reference] | NA |  | 0 [Reference] | NA |
| Hypertension not using medication | 2061 | -0.019 (-0.028, -0.011) | <0.001 |  | -0.030 (-0.042, -0.019) | <0.001 |
| Hypertension using medication | 1537 | -0.006 (-0.015, 0.004) | 0.235 |  | -0.010 (-0.023, 0.003) | 0.124 |
| Longitudinal adherence to anti-hypertensive medication | | | | | | |
| Hypertension, low adherence | 1588 | 0 [Reference] | NA |  | 0 [Reference] | NA |
| Hypertension, moderate adherence | 858 | 0.012 (-0.008, 0.031) | 0.243 |  | 0.007 (-0.010, 0.024) | 0.407 |
| Hypertension, high adherence | 1152 | 0.012 (-0.006, 0.030) | 0.182 |  | 0.022 (0.007, 0.037) | 0.005 |
| Normotension | 5631 | 0.020 (0.007, 0.033) | 0.003 |  | 0.030 (0.019, 0.041) | <0.001 |
| Compared to normotension |  |  |  |  |  |  |
| Normotension | 5631 | 0 [Reference] | NA |  | 0 [Reference] | NA |
| Hypertension, low adherence | 1588 | -0.020 (-0.033, -0.007) | 0.003 |  | -0.030 (-0.041, -0.019) | <0.001 |
| Hypertension, moderate adherence | 858 | -0.008 (-0.025, 0.008) | 0.331 |  | -0.023 (-0.037, -0.009) | 0.002 |
| Hypertension, high adherence | 1152 | -0.008 (-0.022, 0.007) | 0.294 |  | -0.008 (-0.021, 0.004) | 0.203 |

NA, not applicable.

^a^ β coefficient was estimated using linear mixed models, with a positive value representing decelerated cognitive decline in comparison.

Adjusted covariates included age, sex, education, cohabitation status, physical activity, alcohol consumption, current smoking, physical disability, hypertension, diabetes, cancer, chronic lung disease, heart disease, stroke, kidney disease, and baseline measurements of blood pressure (systolic and diastolic blood pressure).

**Table S14.** Baseline characteristics comparison between participants included and excluded from analysis in CHARLS.

| **Characteristics** | **No. (%)** | | |
| --- | --- | --- | --- |
|  | **Excluded**  **n=8479** | **Included**  **n=9229** | ***P* value ^a^** |
| Age, mean (SD), y | 60.1 (11.2) | 57.1 (8.9) | <0.001 |
| Women | 4686 (55.3) | 4542 (49.2) | <0.001 |
| Education |  |  | <0.001 |
| Less than high school | 7479 (88.4) | 8066 (87.4) |  |
| High school or equivalent | 773 (9.1) | 1020 (11.1) |  |
| College and higher | 211 (2.5) | 143 (1.5) |  |
| Living alone | 1379 (16.3) | 881 (9.5) | <0.001 |
| Physical exercise | 1707 (20.1) | 2721 (29.5) | <0.001 |
| Alcohol consumption | 947 (11.2) | 1623 (17.6) | <0.001 |
| Current smoking | 1867 (22.0) | 3004 (32.5) | <0.001 |
| Physical disability | 1623 (19.1) | 1046 (11.3) | <0.001 |
| Hypertension | 3167 (37.4) | 3598 (39.0) | 0.026 |
| Diabetes | 498 (5.9) | 564 (6.1) | 0.526 |
| Cancer | 89 (1.0) | 78 (0.8) | 0.184 |
| Chronic lung disease | 846 (10.0) | 858 (9.3) | 0.131 |
| Heart disease | 1059 (12.5) | 1071 (11.6) | 0.074 |
| Stroke | 297 (3.5) | 189 (2.0) | <0.001 |
| Kidney disease | 483 (5.7) | 496 (5.4) | 0.366 |

Abbreviations: CHARLS, China Health and Retirement Longitudinal Study; SD, standard deviation.

^a^ P value reported for differences between groups using a t-test or chi-squared test.


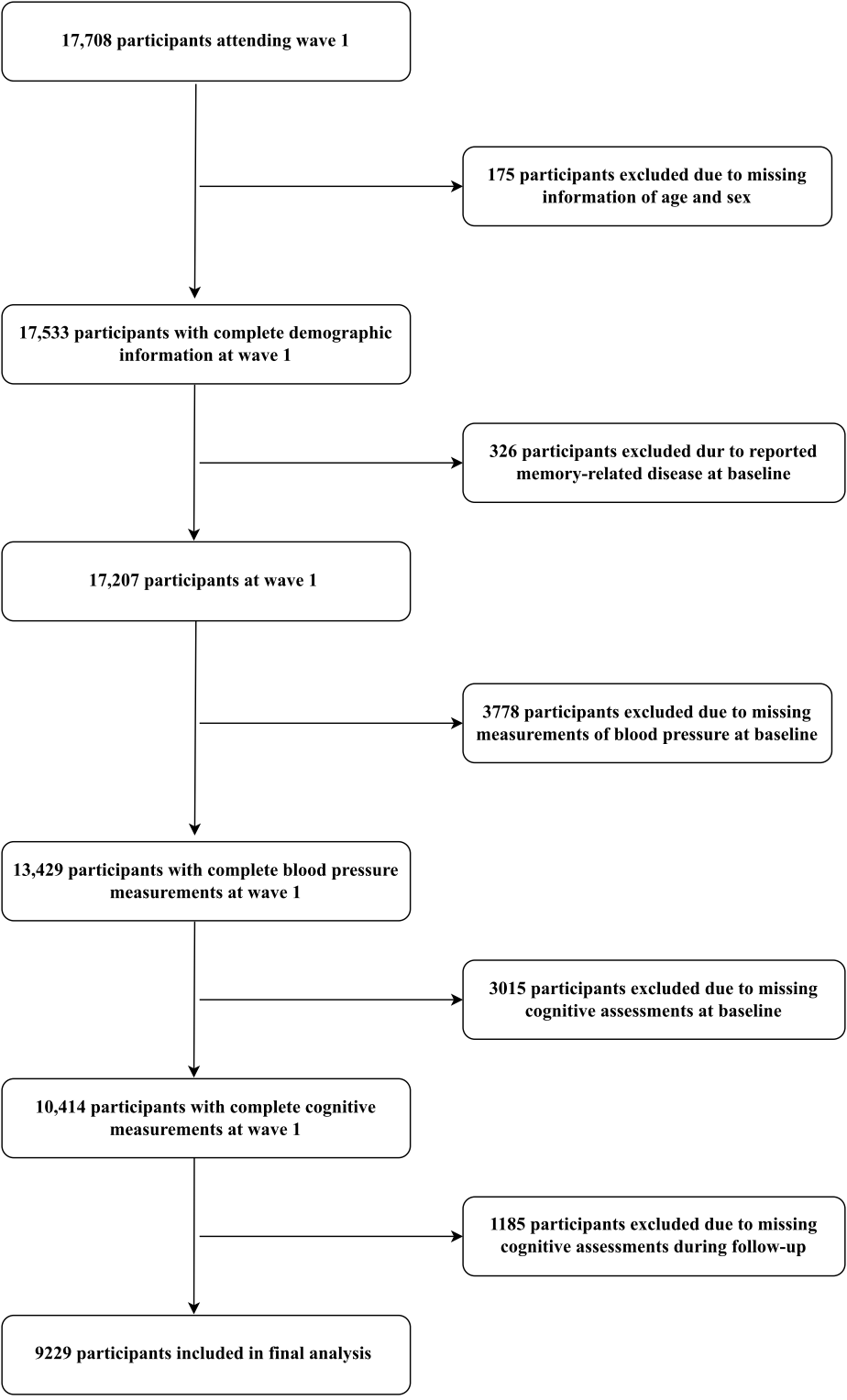


**Figure S1.** Participants selection diagram.


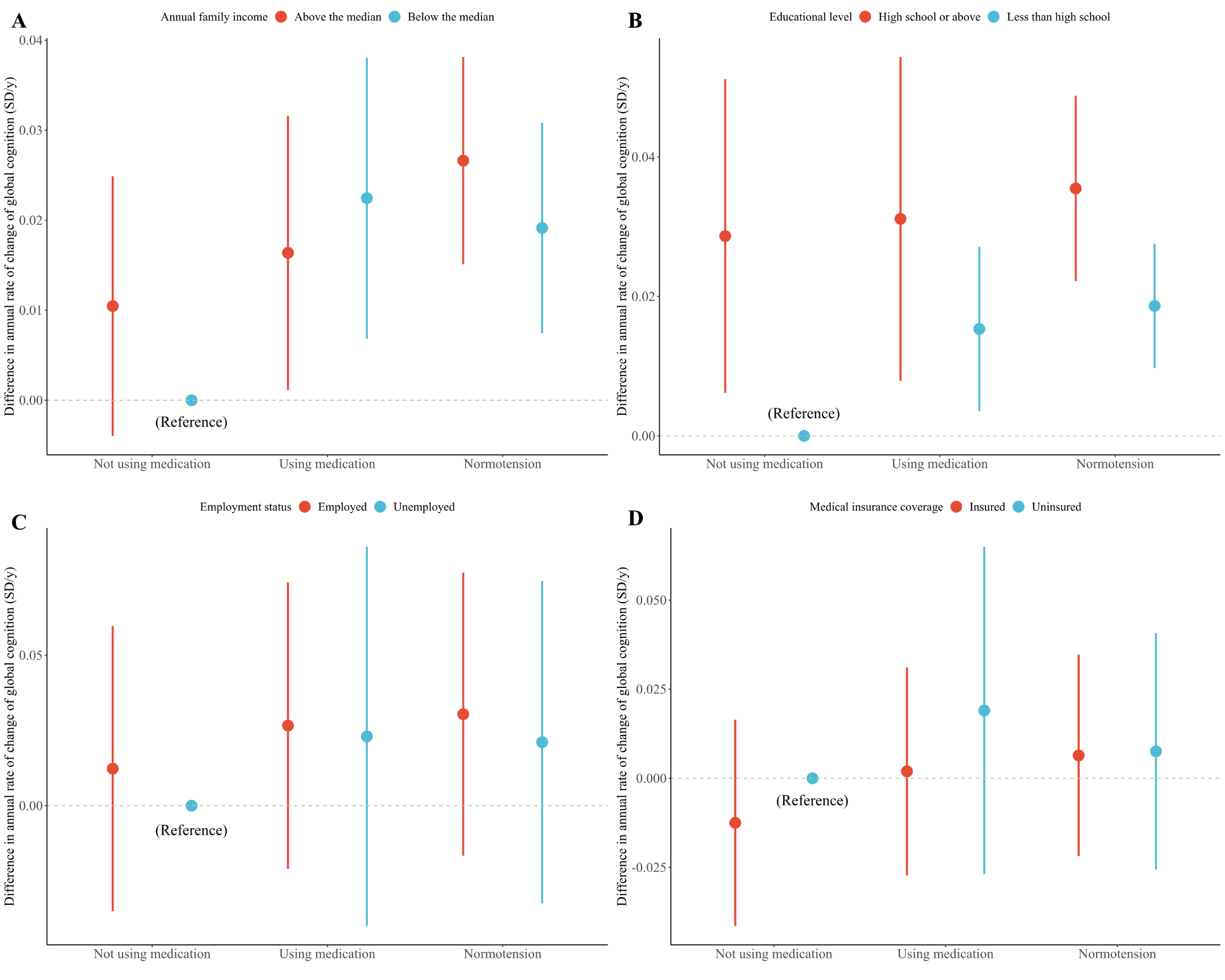


**Figure S2.** Joint associations of baseline anti-hypertensive medication use and socioeconomic status with declines in global cognition.

Joint categories were created by combining baseline anti-hypertensive medication use and socioeconomic factors. Differences in the annual rate of cognitive change were estimated using linear mixed models, controlling for age, sex, cohabitation status, physical activity, alcohol consumption, current smoking, physical disability, hypertension, diabetes, cancer, chronic lung disease, heart disease, stroke, kidney disease, and baseline measurements of blood pressure (systolic and diastolic blood pressure).


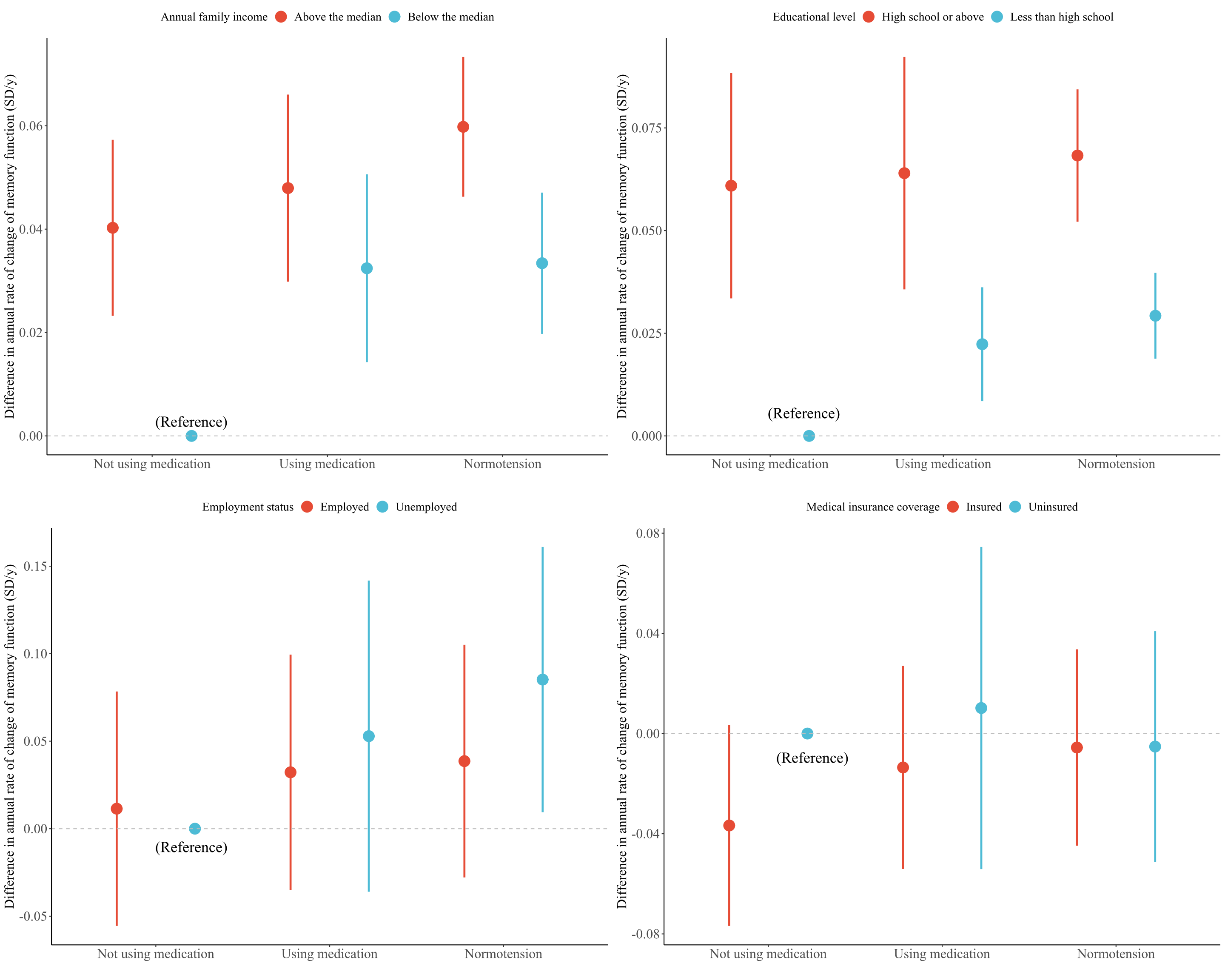


**Figure S3.** Joint associations of baseline anti-hypertensive medication use and socioeconomic status with declines in memory function.

Joint categories were created by combining baseline anti-hypertensive medication use and socioeconomic factors. Differences in the annual rate of cognitive change were estimated using linear mixed models, controlling for age, sex, cohabitation status, physical activity, alcohol consumption, current smoking, physical disability, hypertension, diabetes, cancer, chronic lung disease, heart disease, stroke, kidney disease, and baseline measurements of blood pressure (systolic and diastolic blood pressure).


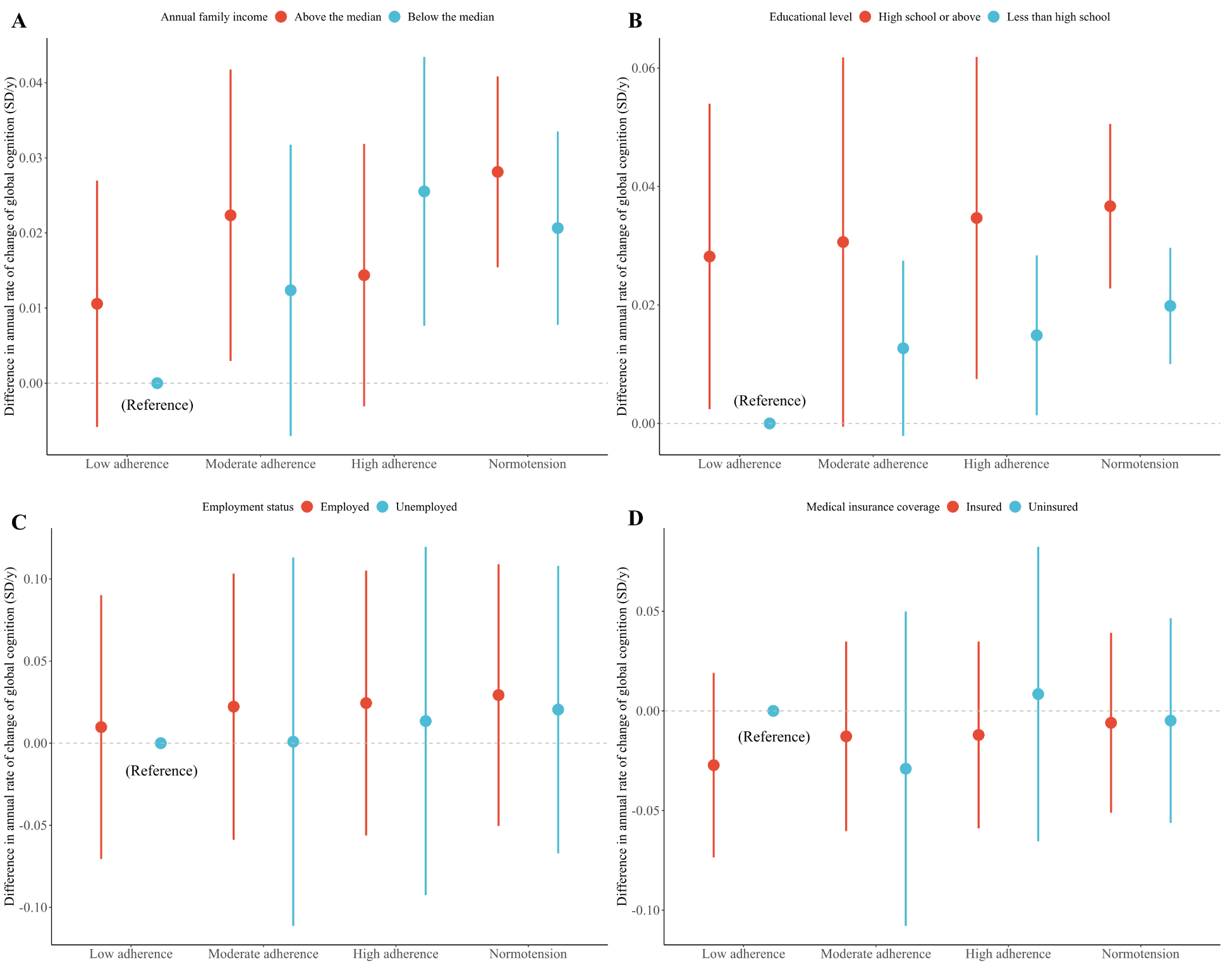


**Figure S4.** Joint associations of longitudinal anti-hypertensive medication adherence and socioeconomic status with declines in global cognition.

Joint categories were created by combining longitudinal anti-hypertensive medication adherence and socioeconomic factors. Differences in the annual rate of cognitive change were estimated using linear mixed models, controlling for age, sex, cohabitation status, physical activity, alcohol consumption, current smoking, physical disability, hypertension, diabetes, cancer, chronic lung disease, heart disease, stroke, kidney disease, and baseline measurements of blood pressure (systolic and diastolic blood pressure).


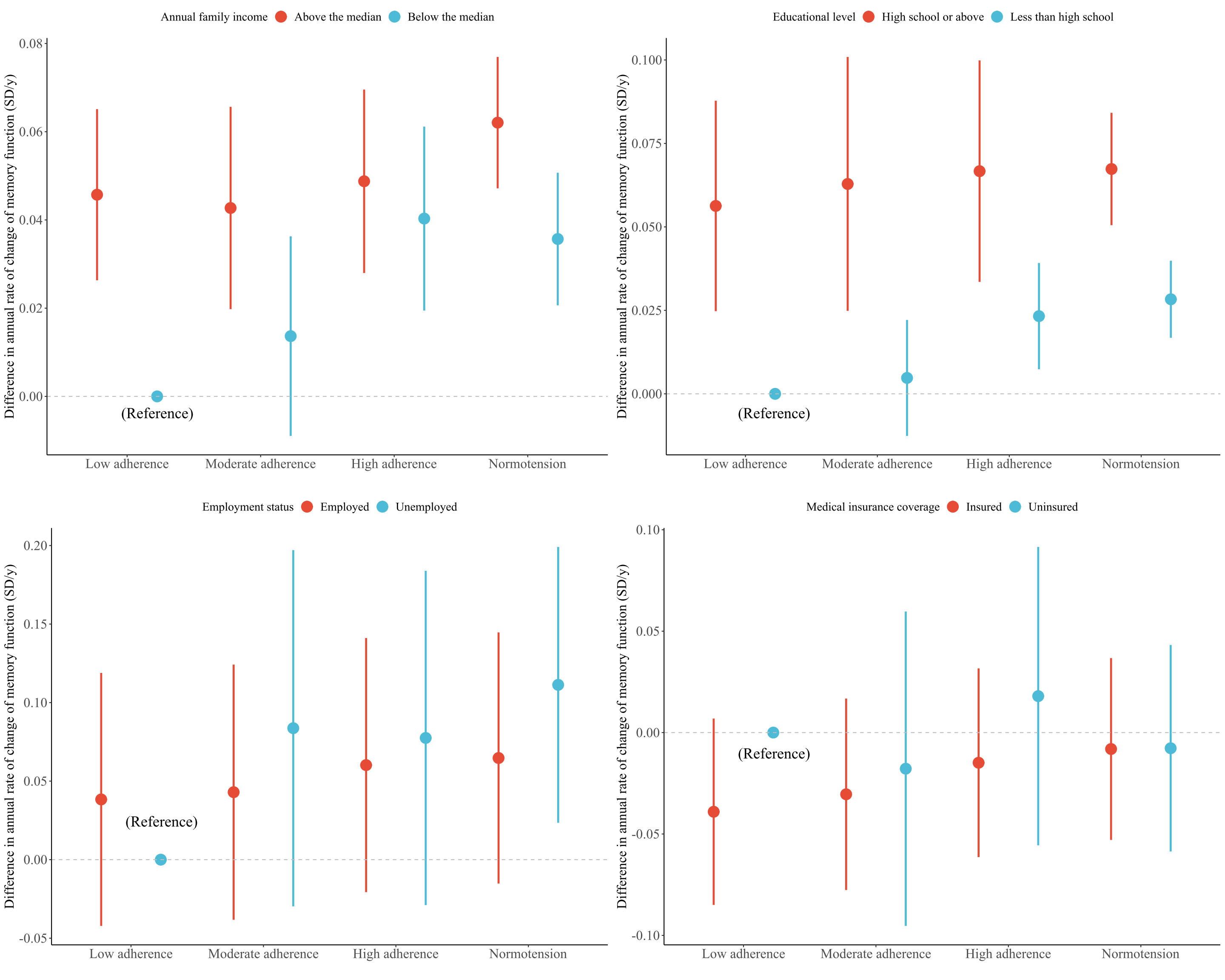


**Figure S5.** Joint associations of longitudinal anti-hypertensive medication adherence and socioeconomic status with declines in memory function.

Joint categories were created by combining longitudinal anti-hypertensive medication adherence and socioeconomic factors. Differences in the annual rate of cognitive change were estimated using linear mixed models, controlling for age, sex, cohabitation status, physical activity, alcohol consumption, current smoking, physical disability, hypertension, diabetes, cancer, chronic lung disease, heart disease, stroke, kidney disease, and baseline measurements of blood pressure (systolic and diastolic blood pressure).


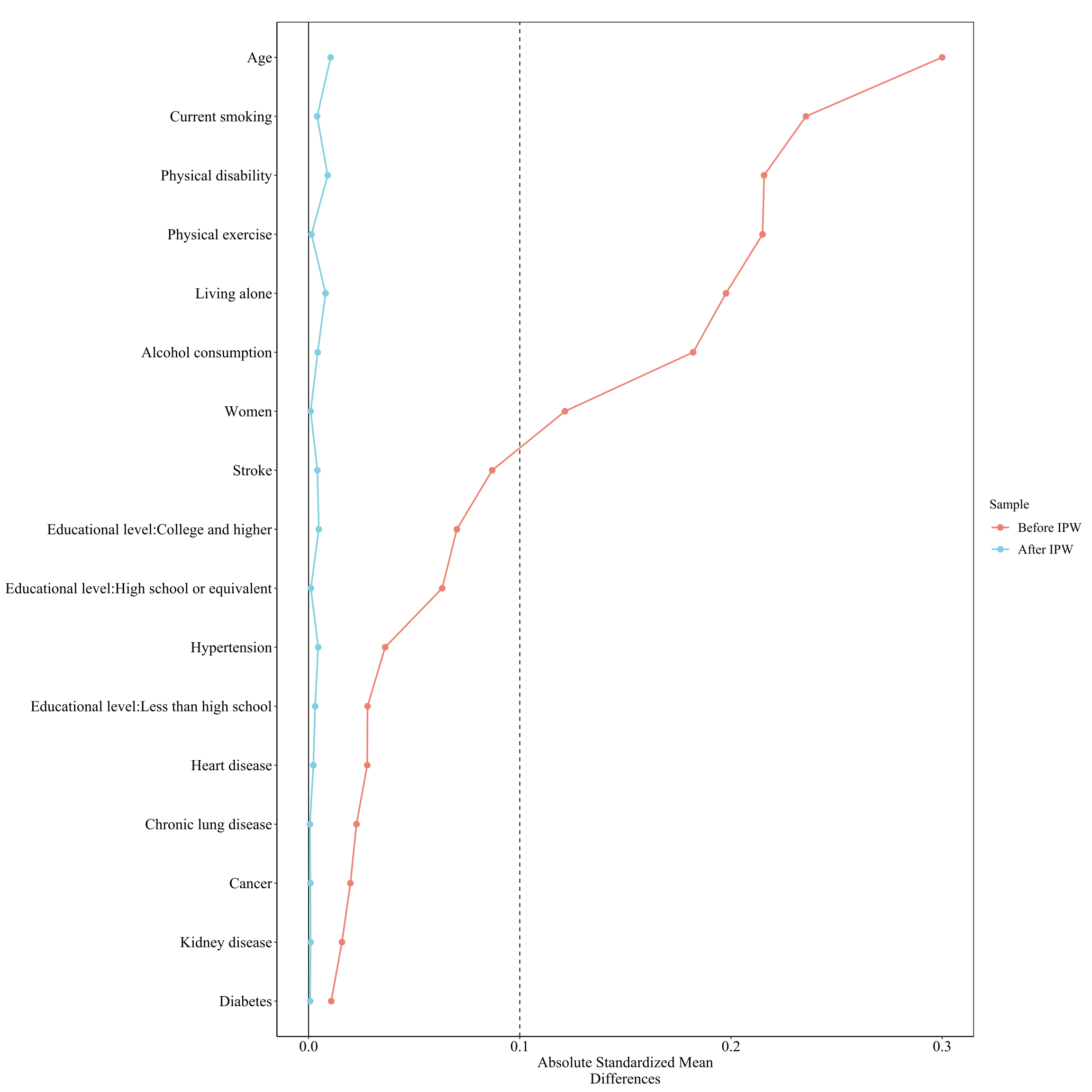


**Figure S6.** Love plot assessing the difference in baseline characteristics between participants included in and excluded from the primary analysis, before and after inverse probability weighting.

IPW, inverse probability weighting.

A binary logistic regression model including baseline variables was applied to estimate the probability of being included in the primary analysis for each participant, with the IPW weights applied to re-weight the sample. An absolute standardized mean difference of 0.1 was used to determine the significance of the imbalance.


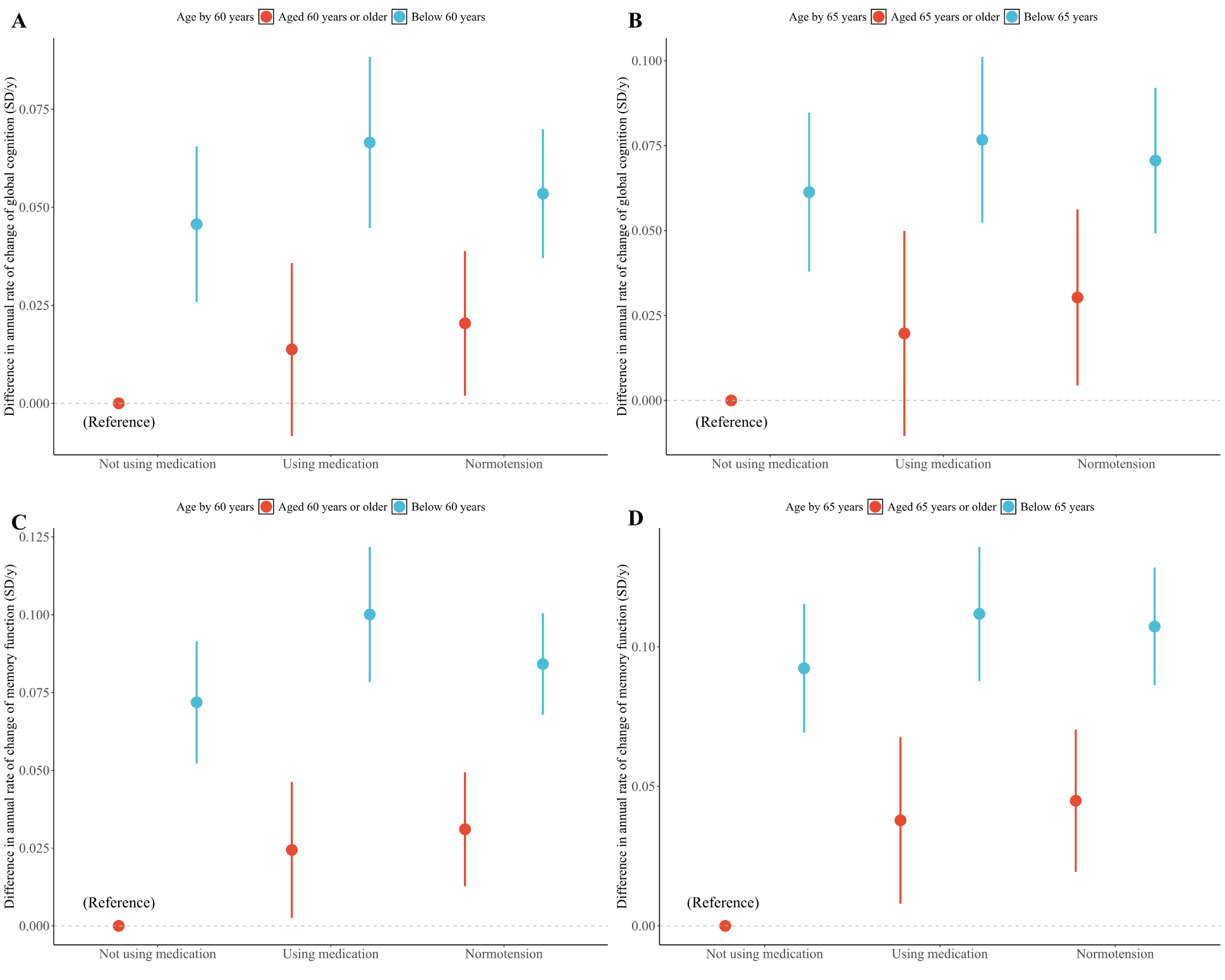


**Figure S7.** Joint associations of baseline anti-hypertensive medication use and baseline age with cognitive aging.

Joint categories were created by combining baseline anti-hypertensive medication use and age groups. Differences in the annual rate of cognitive change were estimated using linear mixed models, controlling for age, sex, education, cohabitation status, physical activity, alcohol consumption, current smoking, physical disability, hypertension, diabetes, cancer, chronic lung disease, heart disease, stroke, kidney disease, and baseline measurements of blood pressure (systolic and diastolic blood pressure).


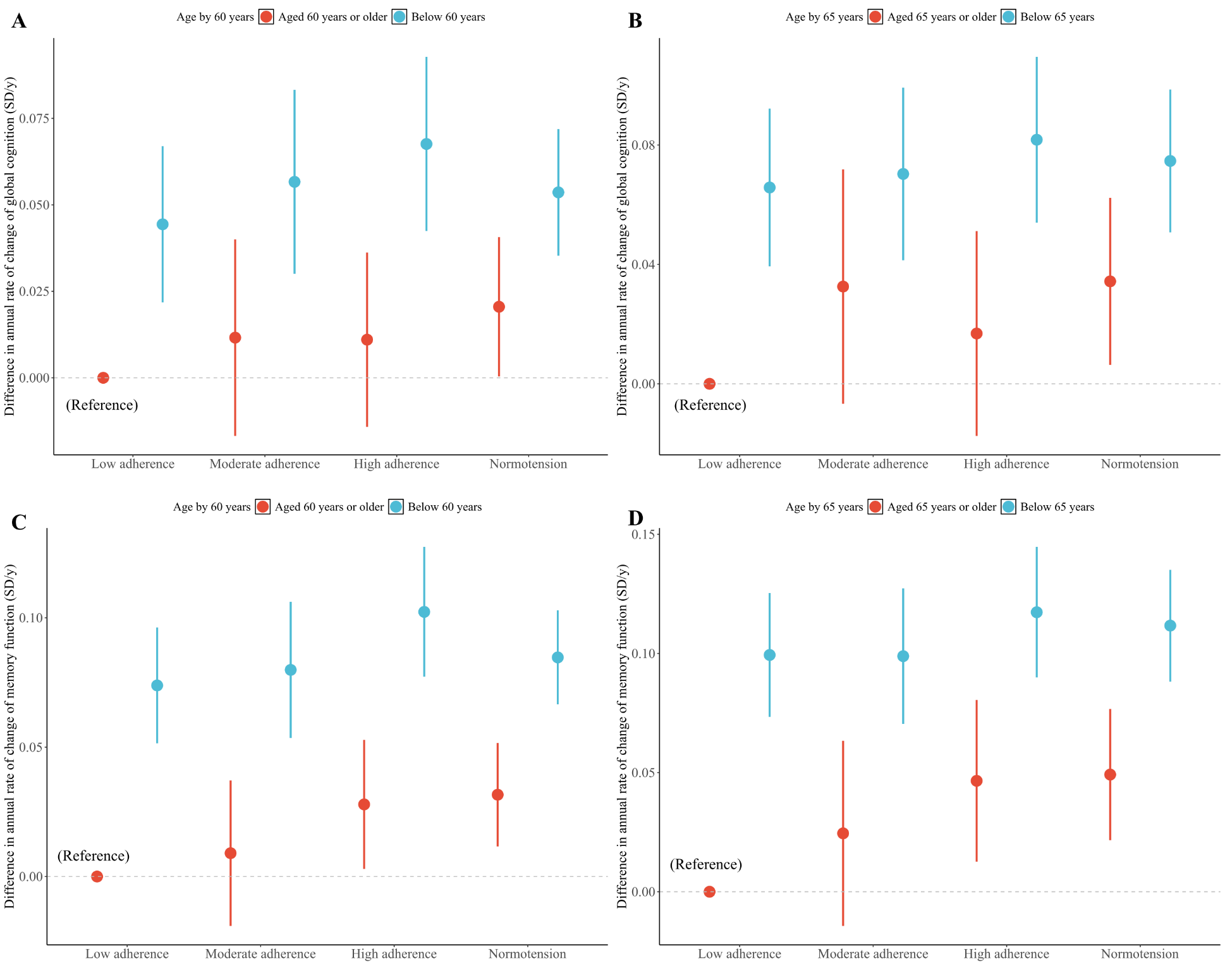


**Figure S8.** Joint associations of longitudinal anti-hypertensive medication adherence and baseline age with cognitive aging.

Joint categories were created by combining longitudinal anti-hypertensive medication adherence and age groups. Differences in the annual rate of cognitive change were estimated using linear mixed models, controlling for age, sex, education, cohabitation status, physical activity, alcohol consumption, current smoking, physical disability, hypertension, diabetes, cancer, chronic lung disease, heart disease, stroke, kidney disease, and baseline measurements of blood pressure (systolic and diastolic blood pressure).
